# Dual hypoxia-responsive supramolecular complex for cancer target therapy

**DOI:** 10.1038/s41467-023-41388-2

**Published:** 2023-09-13

**Authors:** Jian-Shuang Guo, Juan-Juan Li, Ze-Han Wang, Yang Liu, Yu-Xin Yue, Hua-Bin Li, Xiu-He Zhao, Yuan-Jun Sun, Ya-Hui Ding, Fei Ding, Dong-Sheng Guo, Liang Wang, Yue Chen

**Affiliations:** 1 College of Pharmacy, State Key Laboratory of Medicinal Chemical Biology, Tianjin Key Laboratory of Molecular Drug Research, Nankai University, Tianjin, 300353 China; 2 College of Chemistry, State Key Laboratory of Elemento-Organic Chemistry, Key Laboratory of Functional Polymer Materials (Ministry of Education), Nankai University, Tianjin, 300071 China; 3 College of Chemistry, State Key Laboratory of Medicinal Chemical Biology, Nankai University, Tianjin, 300071 China

**Keywords:** Drug development, Drug delivery, Molecular capsules

## Abstract

The prognosis with pancreatic cancer is among the poorest of any human cancer. One of the important factors is the tumor hypoxia. Targeting tumor hypoxia is considered a desirable therapeutic option. However, it has not been translated into clinical success in the treatment of pancreatic cancer. With enhanced cytotoxicities against hypoxic pancreatic cancer cells, BE-43547A2 (BE) may serve as a promising template for hypoxia target strategy. Here, based on rational modification, a BE prodrug (NMP-BE) is encapsulated into sulfonated azocalix[5]arene (SAC5A) to generate a supramolecular dual hypoxia-responsive complex NMP-BE@SAC5A. Benefited from the selective load release within cancer cells, NMP-BE@SAC5A markedly suppresses tumor growth at low dose in pancreatic cancer cells xenograft murine model without developing systemic toxicity. This research presents a strategy for the modification of covalent compounds to achieve efficient delivery within tumors, a horizon for the realization of safe and reinforced hypoxia target therapy using a simple approach.

## Abstract (web summary)

The natural product BE-43547A2 (BE) could potentially serve as a template of hypoxia target strategy for treating pancreatic cancer, but the unsatisfactory pharmacokinetics profile and severe toxicity impeded the application of BE or its derivatives. Here the authors report a supramolecular dual hypoxia-responsive BE-based complex for achieving efficient drug delivery within tumors.

## Introduction

Pancreatic cancer is a leading cause of cancer death worldwide and its global burden has increased dramatically over the past years^1,2^. The latest statistics show that the 5-year overall survival rate of pancreatic cancer is merely 12.5% (https://seer.cancer.gov), which is much lower compared with many other cancer types, rendering it a major medical challenge^2,3^. Pancreatic cancer is commonly characterized by severe hypoxia regions, it represents one of the most hypoxic cancer with < 3 mmHg pO_2_ in portions of tumor tissues, more than a ten-fold decrease compared to normal tissues^4,5^. Hypoxia contributes to the aggressiveness of pancreatic ductal adenocarcinoma (PDAC) by provoking malignant epithelial-mesenchymal transition^6^, enrichment of cancer stem cell population^7^, and strengthened glycolysis^8–10^. Besides, hypoxia predicts aggressive growth in pancreatic cancer xenografts^11^ and the hypoxia-inducible transcription factors, HIF-1α, is considered a predictor of clinical outcome in patients with PDAC^12^. Consequently, hypoxia-targeted therapeutics have emerged as promising options in the precisive treatment of pancreatic cancer^13,14^. Although candidates targeting hypoxia-related proteins (HIF-1α^15–18^ or mTOR^19,20^) or glycolysis^21,22^ proved effective in laboratory investigations or early phase clinical studies, none of them has paved its way into the market as anti-PDAC drug to the best of our knowledge^13,14^.

When O_2_ is limited, certain chemical functional groups have the potential to be metabolized by enzymatic reduction^23^. Bioreductively activated prodrugs converting hypoxia-sensitive cytotoxins to their active forms primarily target DNA^24,25^ and there is already clinical evidence for their activities against pancreatic cancer^24^. Nevertheless, the outcomes of clinical phase III studies on bioreductive prodrugs are disappointing^26^. Besides the limited extravascular penetration of prodrugs, the other key weakness is that their activation is largely dependent on the reductase activity and/or cellular reduction potential in cancer cells. However, there is individual variability among clinical patients, and the clinical efficacy of the bioreductive prodrug cannot be guaranteed^24,26,27^.

Thereafter, hypoxia-selective medications that work through different modes of action are eagerly warranted, not only for therapeutic purposes but for the discovery of hypoxia-related mechanisms underlying the resistance and malignant progression of tumor. However, such compounds are rare. For example, after screening 20,000 different cultivated broths of microorganisms, Ikeda et al. discovered that rakicidin A (a 4-amido-2,4-pentadieneoate (APD) cyclodepsipeptide displayed moderate hypoxia-selective cytotoxicity^28^. In 2017, Poulsen et al. revealed that another type of APD cyclodepsipeptide BE-43547 compounds also exhibited hypoxia-selective cytotoxicity with a significant up to 60-fold decreased IC_50_ value against pancreatic cancer cells (PANC1) under hypoxia compared to normoxia^29^. Subsequently, they made a breakthrough in demonstrating that rather than the regular DNA targeting pattern, APD cyclodepsipeptides induced fast collapse of mitochondrial function and ultrastructural integrity in hypoxic cancer cells^30^. More recently, we synthesized the BE alkynyl probe upon which we disclosed that BE covalently binds the cysteine234 residue of eukaryotic translation elongation factor 1 alpha 1 (eEF1A1) to exert its anti-pancreatic cancer effects^31^. Following this, eEF1A1 was found highly expressed under hypoxia in pancreatic cancer cells^32^ and acts a vital role in regulating the stemness of pancreatic cancer cells^31^. Besides, utilizing 99 clinical specimens of pancreatic cancer patients, eEF1A1 protein levels are found positively correlated with pancreatic cancer stage but negatively correlated with patient survival^31^. This highlights the importance of eEF1A1 in the progression of pancreatic cancer. Specifically targeting eEF1A1, BE may serve as a promising lead for hypoxia target therapy in pancreatic cancer treatment.

We have worked out synthetic routes that could supply BE and other structure similar natural products or their derivatives^33–39^ for in vivo experiments^29,40–44^ refers to the synthetic study of other researches. However, low water solubility and toxicity of BE limited its further application as anticancer reagent and there is a clear need to develop a functional formulation of BE to reduce its side effects and improve its anticancer effects. As a supramolecular carrier, calixarenes have been used as a molecular vessel to transport therapeutic medications into tumors, thereby enhancing therapeutic efficacy and/or alleviating side effects^45–47^. In contrast to conventional nanoscale drug-delivery systems, calixarenes feature well-defined molecular structures and weight as well as operational simplicity, which could assure batch-to-batch consistency through rigorous manufacturing procedures^46–50^. Moreover, the unusual properties of tunable cavity size and convenient modification empower these macrocyclic hosts with intriguing molecular recognition ability, thereby quantitatively binding a variety of drug guests^50–52^. In our issue, an ideal calixarene host should own the following specific characteristics: (i) high binding affinity between host and BE to prevent unwanted leaking^45,53^, and (ii) efficient delivery to tumors by targeting the characteristics of tumor microenvironment^50,54–57^. As a result, the key challenge emerges to be designing promising calixarene macrocycles with good water solubility, excellent biocompatibility, excellent binding affinity to guest molecules and targeting ability.

**Fig. 1:**
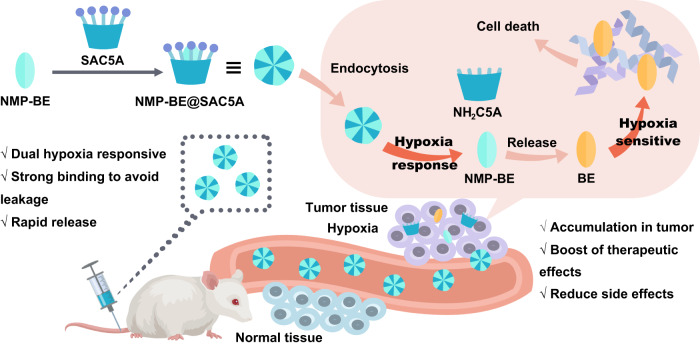
Conceptual illustration of the dual hypoxia-responsive supermolecular complex for cancer therapy. The supermolecular prodrug NMP-BE@SAC5A is formed by the host-guest complexation of hypoxia-responsive molecular container SAC5A with NMP-BE. The azo groups of SAC5A quickly respond under hypoxic condition, leading to the release of loads within tumors. NMP-BE can subsequently release hypoxia-sensitive toxin BE to exert anticancer effects.

In this work, we proposed a fusion of covalent and non-covalent drug delivery strategy for the treatment of pancreatic cancer. The active and susceptible covalent binding site of BE was temporarily masked by N-methyl-piperizine via a 1,6-conjugate addition to generate an amine-adducted prodrug N-methyl-piperizine-BE-43547A_2_ (NMP-BE). With similar inhibitory activities against PANC-1 cells and reduced toxicity against normal cells at certain concentrations. NMP-BE is further capable of being encapsulated non-covalently into a complex with sulfonated azocalix[5]arene (SAC5A). The azo groups of azocalixarene are hypoxia-responsive^50,54^, and can be reduced by overexpressed azoreductases in hypoxic cancer cells. SAC5A can promote the accumulation of payload toward the tumors. When NMP-BE is unloaded within cancer cells, it could subsequently release the hypoxia-sensitive toxin BE to achieve a host-guest dual hypoxia-responsive therapeutic purpose (Fig. 1). This innovative formulation was tested in vitro and further in vivo using a PANC-1 xenograft murine model. NMP-BE@SAC5A significantly suppressed tumor growth and boosted the antitumor efficacy of NMP-BE, accompanied by no systemic toxicity observed.

## Results

### Design and preparation of NMP-BE, SAC5A and NMP-BE@SAC5A

**Fig. 2:**
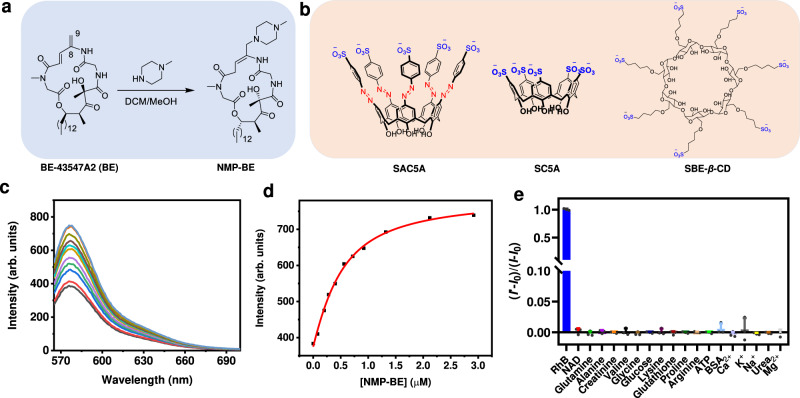
The characterizations of NMP-BE@SAC5A. **a** Synthesis of NMP-BE. **b** The structures of SAC5A, SC5A and SBE-*β*-CD. **c** Competitive fluorescence titration of RhB@SAC5A (0.30/0.50 μM) with NMP-BE (up to 2.9 μM) in PBS (10 mM, pH = 7.4) at 25 °C, *λ*_ex_ = 554 nm. **d** The associated titration curve at *λ*_em_ = 575 nm and fit according to a 1:1 binding stoichiometry. **e** Fluorescent responses of RhB@SAC5A (10/10 μM) upon the addition of various biologically coexisting species in blood. Data are presented as mean ± SD (*n* = 3 biologically independent samples). Source data are provided as a Source Data file.

We formerly used N-methyl-piperizine (NMP) for the 1,4-conjugate addition on ovatodiolide and its derivative^58^. In phosphate-buffered saline (PBS) solution, the nascent prodrug provides appropriately sustained release of active parent compound. Temporarily masking the liable *α, β*-unsaturated lactone significantly enhanced the stability toward human liver microsomes^58^ or PK profile^59^. According to previous study^31,33,42^, the C8-C9 double bond of BE was supposed to covalently react with the Cys234 of its target (eEF1A1) protein via a 1,6-conjugate addition. We audaciously mimicked this process by subjection of NMP directly into BE or its penultimate ethyl sulfonyl ester crude, where 1,6-conjugate addition occurred smoothly to deliver a more soluble prodrug NMP-BE (Fig. 2a, and Supplementary Figs. 3–6). The in vitro assays suggested that NMP-BE could release BE in HEPES buffer (Supplementary Fig. 7). The preliminary PK results indicated a rapid release of NMP-BE to BE post caudal vein injection. Notably, in a 72 h MTT cytotoxic assay, the potency of NMP-BE (IC_50_ = 0.65 μM) against PANC1 cells is comparable to that of BE (IC_50_ = 0.72 μM). In this assay, we used NMP to benchmark, the results indicated NMP is non-cytotoxic at concentrations ranging from 0 to 40 μM (Supplementary Fig. 8). Furthermore, as shown in Supplementary Fig. 9, NMP-BE also exhibited hypoxia-selective cytotoxicity in PANC1 cells, and the IC_50_ value of hypoxia (0.034 μM) is significantly lower than that of normoxia (1.38 μM).

Moreover, in a preliminary acute toxicity experiment, mice can tolerate caudal vein injection of NMP-BE fumaric acid at dosages up to 25 mg/kg, no animal death or fracture of tail occurred though darkened tails and crust were observed on the injection sites (Supplementary Fig. 10). The pharmacokinetic study (10 mg/kg iv) of NMP-BE indicated AUC parameter ≈ 4941 ng/mL*h and t_1/2_ = 5.82 h (Supplementary Fig. 11). To improve its anticancer effects, we turned our attention on the formulation of BE, especially calixarenes for the efficient delivery of BE toward tumor tissues.

A weak binding between BE and calixarene will enable premature separation when the complex is exposed to body fluid dilution, leading to nondifferential death of both malignant and healthy cells^53^. Considering the hydrophobic nature of BE as well as its bulky size caused by its lipid chain and macrocycle, encapsulation of BE in the cavity of calixarene may be problematic. It is indicated that the distal amine atom of NMP group in NMP-BE could be protonated under physiological pH conditions^60^. The generated cation could be utilized to form an electrostatic interaction with the upper-rim functionalities of calixarene. Therefore, we made efforts in trimming the calixarene to ensure host-guest strong binding affinity.

Considering the low water solubility of BE, a soluble container was necessary for the complex to achieve systemic delivery. After extensive attempts, we designed and synthesized SAC5A endowed with a longitudinally deep cavity suitable for hydrophobic guest incorporation (Supplementary Figs. 1, 2)^50^. The sulfonate groups at the upper rim, which exist in the form of anions under physiological pH conditions, are designed for the enhanced interaction with NMP-BE, as well as to refine the water solubility of the host-guest complex^61^. In the subsequent assays, three vectors (SAC5A, sulfonated calix[5]arene (SC5A) and sulfobutyl ether-*β*-cyclodextrin (SBE-*β*-CD)) were tested for comparison (Fig. 2b). We investigated the capacity of SAC5A to bind to NMP-BE. By fitting according to a 1:1 competitive binding stoichiometry, SAC5A showed a *K*_a_ value of (5.1 ± 0.5) × 10^6 ^M^−1^ (Fig. 2c, d and Supplementary Fig. 12) indicating an excellent inclination ability to NMP-BE. The host-guest interactions were also determined by nuclear overhauser effect spectroscopy (Supplementary Fig. 13). Contrary to this event, the shallow-caved SC5A and SBE-*β*-CD had almost no bonding ability to NMP-BE (Supplementary Figs. 14, 15). This illustrated that deep cavitand is necessary to encapsule NMP-BE.

Next, excess sodium dithionite (SDT, a chemical mimic of azoreductase) was added into the SAC5A solution to explore the hypoxic response of SAC5A. Accordingly, SAC5A lost its signature yellow tint due to the complete reduction of diazo groups after adding SDT. The reductive half-life of SAC5A was calculated as 36 s^62^. Therefore, SAC5A can respond quickly under specific stimulus which is a prerequisite to achieving rapid and accurate unloading of drugs. To investigate the release kinetics of NMP-BE, rhodamine B (RhB) was employed as a model fluorescent probe encapsulated in SAC5A^63,64^. The binding affinity was determined to be 1.2 × 10^6 ^M^−1^^62^, which was in the same order of magnitude to that of NMP-BE@SAC5A ((5.1 ± 0.5) × 10^6 ^M^−1^). After adding SDT to the solution of RhB@SAC5A, the fluorescence intensity of RhB was incrementally recovered with a half-life of 14 s^62^. The fast kinetics guaranteed SAC5A to achieve selective and rapid cargo release as desired when exposed to stimulus under hypoxia, establishing the premise for tumor accumulation^65–67^.

Because supramolecular host-guest pairings are susceptible to suffering from undesired competition from biomolecules in organisms, it is essential to evaluate the anti-interference ability of the loaded SAC5A. The experimental results showed that no remarkable fluorescence regeneration of RhB@SAC5A was observed when its solutions were incubated with multiple biomolecules in blood, including nicotinamide adenine dinucleotide (NAD), glutamine, alanine, creatinine, valine, glycine, glucose, lysine, glutathione, proline, arginine, adenosine triphosphate (ATP), bovine serum albumin (BSA), Ca^2+^, K^+^, Na^+^, urea, and Mg^2+^ (Fig. 2e). These results collectively confirmed the robust stability of the loaded SAC5A. Considering the stronger binding affinity of NMP-BE@SAC5A than that of RhB@SAC5A, SAC5A may keep its structural integrity and prevent the inappropriate release of NMP-BE during blood circulation.

### In vitro hypoxia response of SAC5A

**Fig. 3:**
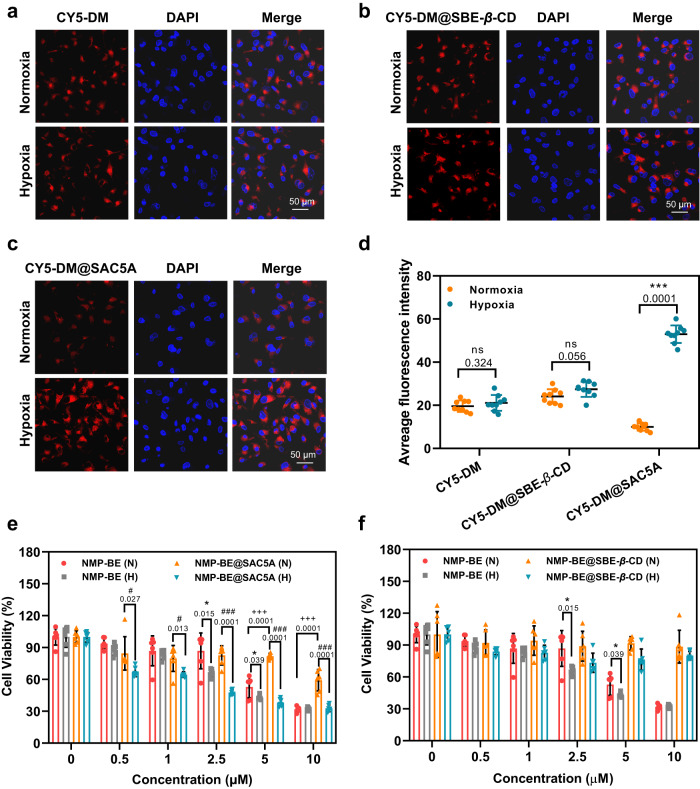
The hypoxia response and cytotoxicity of NMP-BE, NMP-BE@SBE-*β*-CD and NMP-BE@SAC5A. CLSM images of PANC1 cells after treatment with CY5-DM (**a**), CY5-DM@SBE-*β*-CD (**b**) and CY5-DM@SAC5A (**c**) under normoxic and hypoxic conditions and DAPI staining. Scale bar, 50 μm. This was repeated independently for 3 times with similar results. **d** Quantitation of the CLSM micrographs (*n* = 9 biologically independent samples); *P* values are calculated by paired *t* test: ^***^P < 0.001 versus CY5-DM@SAC5A-normoxia group). Cell viabilities of PANC1 cells treated with various concentrations of NMP-BE, NMP-BE@SAC5A (**e**) and NMP-BE@SBE-*β*-CD (**f**) under normoxic (N) and hypoxic conditions (H) (*n* = 6 biologically independent samples). Data are presented as mean ± SD. *P* values are calculated by paired *t* test: ^*^P < 0.05 versus NMP-BE-normoxia group; ^#^ *P* < 0.05, ^###^ *P* < 0.001 versus NMP-BE@SAC5A-normoxia group; ^+++^*P* < 0.001 versus NMP-BE-normoxia group. Source data are provided as a Source Data file.

To verify the in vitro hypoxic response of SAC5A, 1,1’,3,3,3’,3’-hexamethylindodicarbocyanine (CY5-DM, a commercially available and biocompatible dye that can be complexed by SAC5A with an intense fluorescence quenching) was chosen to be the imaging probe and preloaded into SAC5A to form CY5-DM@SAC5A. In this assay, SBE-*β*-CD was employed to generate CY5-DM@SBE-*β*-CD as a benchmark. We compared the fluorescence imaging ability of CY5-DM (10 μM), CY5-DM@SBE-*β*-CD (10/10 μM), and CY5-DM@SAC5A (10/10 μM) using confocal laser scan microscopy (CLSM) in living cells under normoxic and hypoxic conditions. As depicted in Fig. 3c, under normoxic conditions, the PANC1 cells treated with CY5-DM@SAC5A showed lower red fluorescence intensity compared with that in hypoxic conditions. At the same time, no significant difference in fluorescence intensities was observed within the CY5-DM and CY5-DM@SBE-*β*-CD treated groups (Fig. 3a, b). Further quantitative analysis (Fig. 3d) indicated that the average fluorescence intensity in PANC1 cells upon CY5-DM@SAC5A treatment under hypoxia was ~5.3-fold of that under normoxia. The increased fluorescence manifested the hypoxia-selective release performance of the supramolecular complex. Then the cellular uptake mechanisms of SAC5A were evaluated, its endocytosis was mainly energy dependent, as pretreatment under 4 °C inhibited cellular uptake of CY5-DM@SAC5A (Supplementary Figs. 16, 17) and the fluorescent CY5-DM was observed in the entire cytoplasm of the cells, including lysosomes (Supplementary Fig. 18).

We next assessed the cytotoxicity of NMP-BE, SAC5A, and NMP-BE@SAC5A using a 24 h CCK8 assay (6 h reagents treatment followed culture medium replaced and 18 h incubation) under normoxic (5% O_2_) and hypoxic (1% O_2_) conditions. SBE-*β*-CD and NMP-BE@SBE-*β*-CD were used as negative controls. The corresponding results indicated that SAC5A was very safe: its cytotoxicities at concentrations ranging from 10 μM to 0.40 mM were negligible to PANC1 cells under both normoxic and hypoxic conditions (Supplementary Fig. 19). Meanwhlie, the PANC1 cell viability under hypoxia was slightly lower than that under normoxia when treated with NMP-BE at concentrations of 2.5 and 5.0 μM. In contrast, the viable cells in NMP-BE@SAC5A treated group (concentrations of 2.5, 5.0, and 10 μM) dropped to around half under hypoxia compared to normoxia. Notably, in doses of 5.0 and 10 μM, when compared to NMP-BE, NMP-BE@SAC5A exhibited comparable inhibitory activities under hypoxia while declined activities under normoxia (Fig. 3e). In contrast, there were no noticeable difference in the viability of cells when they were treated with NMP-BE@SBE-*β*-CD under normoxic and hypoxic conditions (Fig. 3f). Further, to confirm whether NMP-BE or NMP-BE@SAC5A could reduce the toxicity of BE to normal cells, the cell viabilities of BE, NMP-BE and NMP-BE@SAC5A against human pancreatic nestin expressing (HPNE) cells were measured. As shown in Supplementary Fig. 20, compared with BE and NMP-BE-treated group, the cell viability of NMP-BE@SAC5A-treated group enhanced significantly at concentrations of 2.5 and 5 μM. These results above suggested that SAC5A could respond to hypoxic stimuli to release NMP-BE and the NMP-BE@SAC5A complex may alleviate the toxic effects on normal cells.

### In vivo hypoxia response of SAC5A

**Fig. 4:**
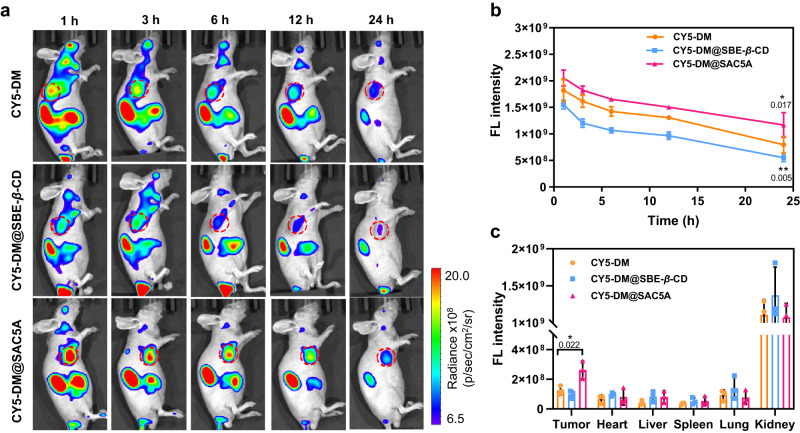
In vivo fluorescence imaging of CY5-DM@SAC5A. **a** In vivo fluorescence images of PANC1-bearing mice after 1, 3, 6, 12, and 24 h intravenous injection of CY5-DM, CY5-DM@SBE-*β*-CD and CY5-DM@SAC5A (*n* = 3 mice per group). Tumor tissues were circled by red solid line. **b** The fluorescence intensity (FL intensity) of the tumor at each time point in vivo was quantitatively analyzed. Data are presented as mean ± SD (*n* = 3 biologically independent samples per group). *P* values are calculated by two-way ANOVA with Turkey’s multiple comparisons test: ^*^P < 0.05, ^**^P < 0.01 versus CY5-DM-treated group; **c** The fluorescence intensity (FL intensity) of the major organs *in* ex vivo at 24 h post injection. Data are presented as the mean ± SD (*n* = 3 biologically independent samples per group). *P* values are calculated by paired *t* test: ^*^P < 0.05 versus CY5-DM^-^treated group. Source data are provided as a Source Data file.

To further investigate the capability of SAC5A for tumor targeting, PANC1 tumor-bearing mice were injected intravenously with free CY5-DM, CY5-DM@SBE-*β*-CD, and CY5-DM@SAC5A. Then, the fluorescence of CY5-DM was recorded at various time points. Cyanine dyes can be distributed in many organs due to their non-targeting nature^68,69^. As shown in Fig. 4a, the mice treated with CY5-DM, CY5-DM@SBE-*β*-CD and CY5-DM@SAC5A exhibited a fluorescence distribution overspread their bodies. The fluorescence signals in the tumors treated with CY5-DM@SAC5A were significantly higher than that of CY5-DM and CY5-DM@SBE-*β*-CD groups at 1 h post injection. And the fluorescence intensities of the tumors treated with CY5-DM@SAC5A were always higher than that of other groups over time post injection (Fig. 4b), suggesting that SAC5A was capable of facilitating the delivery of CY5-DM or boosting their aggregation in tumors. Further quantitative analysis of the ex vivo images at 24 h post injection (Supplementary Fig. 21 and Fig. 4c) indicates that the average fluorescence intensity of the tumor from mice treated with CY5-DM@SAC5A was nearly one-fold higher than mice treated by free CY5-DM. However, there was no significant difference in fluorescence intensity between CY5-DM@SBE-*β*-CD-treated and free CY5-DM-treated group. These results demonstrated that SAC5A may serve as a potential supramolecular carrier which could promote tumor-targeting delivery of NMP-BE to achieve boost of therapeutic efficacy.

### In vivo anticancer effects of NMP-BE@SAC5A

**Fig. 5:**
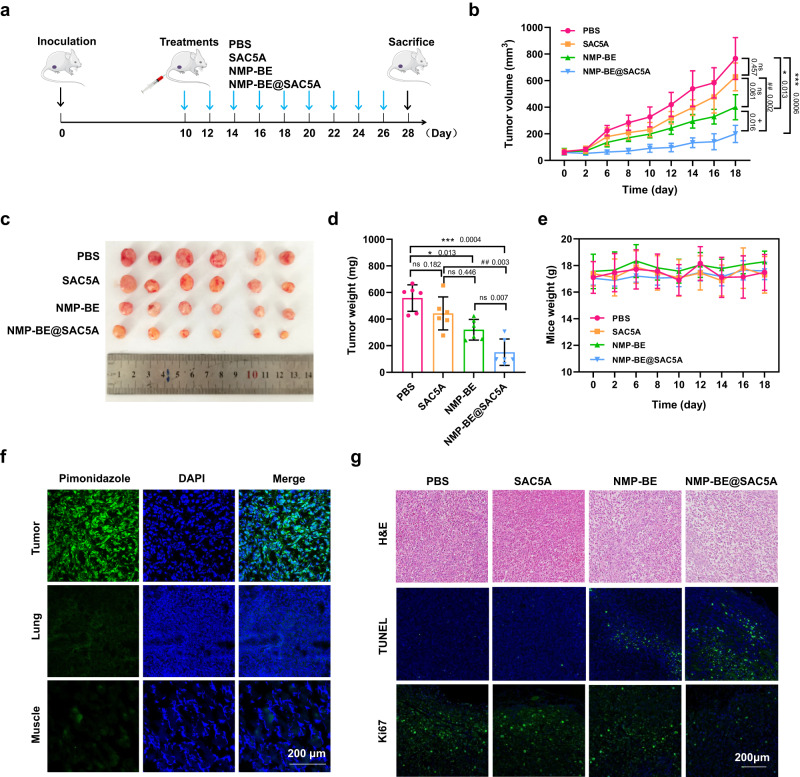
In vivo anticancer effects of NMP-BE@SAC5A. **a** Treatment schema of PANC1 tumor-bearing BALB/c mice after treatment with PBS, NMP-BE (5 mg/kg), SAC5A (15 mg/kg) and NMP-BE@SAC5A (20 mg/kg). **b** Average tumor volume growth curves of the mice treated with PBS, SAC5A, NMP-BE, and NMP-BE@SAC5A. Data are presented as mean ± SD (*n* = 6 mice per group). *P* values are calculated by two-way ANOVA with Turkey’s multiple comparisons test: ns no significance; ^*^P < 0.05, ^***^P < 0.001 versus PBS group; ^##^*P* < 0.01 versus SAC5A group; ^+^*P* < 0.05 versus NMP-BE group. **c** Images of tumors dissected from each mouse in different groups on the day mice were sacrificed. **d** Tumor weight in different groups on the day mice were sacrificed. Data are presented as mean ± SD (*n* = 6 biologically independent samples per group). *P* values were calculated by one-way ANOVA with Turkey’s multiple comparisons test: ns no significance; ^*^P < 0.05, ^***^P < 0.001 versus PBS group; ^##^*P* < 0.01 versus SAC5A group. **e** Curves of average mouse weight in different groups after the day of PBS, SAC5A, NMP-BE and NMP-BE@SAC5A administration. Data are presented as mean ± SD (*n* = 6 mice per group). **f** Pimonidazole staining of tumor, lung and muscle was conducted when the tumor volume was around 50 mm^3^ on day 10 of inoculation. Representative images from *n* = 3 mice. **g** Histological observation of the dissected tumor tissues of different groups. The tumor sections were stained with H&E, TUNEL, and Ki67. Cell nucleus were stained with DAPI. Scale bar, 200 μm. Representative images from *n* = 6 mice per group. Source data are provided as a Source Data file.

We testified the tumor hypoxia at the early stage in PANC1-bearing BALB/c nude mice, the pimonidazole-staining was conducted when the tumor volume was around 50 mm^3^ on day 10 of inoculation. Pimonidazole HCl (Hypoxuprobe-1, 60 mg/kg) was injected intraperitoneally 1 h before the mice were killed. The tumor, lung, and muscle were collected and the immunofluorescence analysis was performed. As shown in Fig. 5f, the intensity of pimonidazole staining was significantly increased in tumor tissue but not in lung and muscle, indicating that there is obvious tumor hypoxia at early stage of the xenograft model.

To further demonstrate the application of SAC5A as a supramolecular carrier for cancer treatment, we investigated the anticancer effect of NMP-BE@SAC5A in PANC1-bearing BALB/c nude mice. For in vivo anticancer efficacy study, 1 × 10^6^ PANC1 cells were subcutaneously injected into the right flank of BALB/c nude mice. After the tumor grows to a suitable size, the tumor was divided into small pieces and then subcutaneously inoculated into the right flank of other mice. After 10 d of inoculation, when the tumor volumes of mice were around 50 mm^3^, PANC1-bearing BALB/c nude mice were separated randomly with a total of six mice in each group. The mice were then administered intravenous doses of PBS, SAC5A (15 mg/kg), NMP-BE (5 mg/kg), and NMP-BE@SAC5A (20 mg/kg) through tail veins every other day for a period of 18 days (Fig. 5a). As illustrated in Fig. 5b and Supplementary Fig. 22, NMP or SAC5A alone had no anticancer effect. The NMP-BE@SAC5A group proved the most suppression in cancer development, which significantly enhanced the anticancer effect of NMP-BE, the therapeutic benefits of this medication became apparent on day 6 following the initial injection. On day 18 after injection, the mice were euthanized, and their tumors were collected. The results showed the prominent in vivo anticancer effects of NMP-BE@SAC5A (Fig. 5c, d), with average tumor weight decreased by ~70% compared with the PBS-treated group. The mice treated with NMP-BE alone exhibited limited therapeutic effects, with tumor weight decreased by ~30%. Notably, darkened tails or crust were observed in the NMP-BE-treated group, while these were relieved in the NMP-BE@SAC5A-treated group visually. During the 18-day period, the mice weight remained stable (Fig. 5e), indicating that those treatments were well tolerated. In addition, we also investigated the anticancer effects of NMP-BE@SBE-*β*-CD. However, NMP-BE@SBE-*β*-CD did not further enhance the anticancer effects of NMP-BE (Supplementary Fig. 23).

The hematoxylin-eosin (H&E) staining and terminal deoxynucleotidyl transferase dUTP nick end labeling (TUNEL) tests were next carried out to evaluate the cell pathology changes and apoptosis in the tumors of every groups. The greatest morphological alterations, such as condensed and hyperchromatic nuclei, were produced in the tumor tissues of NMP-BE@SAC5A-treated group, as shown by the findings of H&E staining (Fig. 5g). Consistent with this, the most positive TUNEL signal intensities were observed in the tumors of NMP-BE@SAC5A-treated group, which firmly indicated that SAC5A boosted the anticancer effects of NMP-BE in vivo, while negligible apoptosis was observed in the PBS- and SAC5A-treated groups. Meanwhile, we verified the effects of different treatments on cancer cell proliferation by immunofluorescence Ki67 staining. The NMP-BE@SAC5A-treated group resulted in a more distinct decrease in the number of Ki67 positive tumor cells compared with that of other groups, demonstrating that NMP-BE@SAC5A markedly restrained proliferation of cancer cells.

### Biosafety evaluation of NMP-BE@SAC5A

**Fig. 6:**
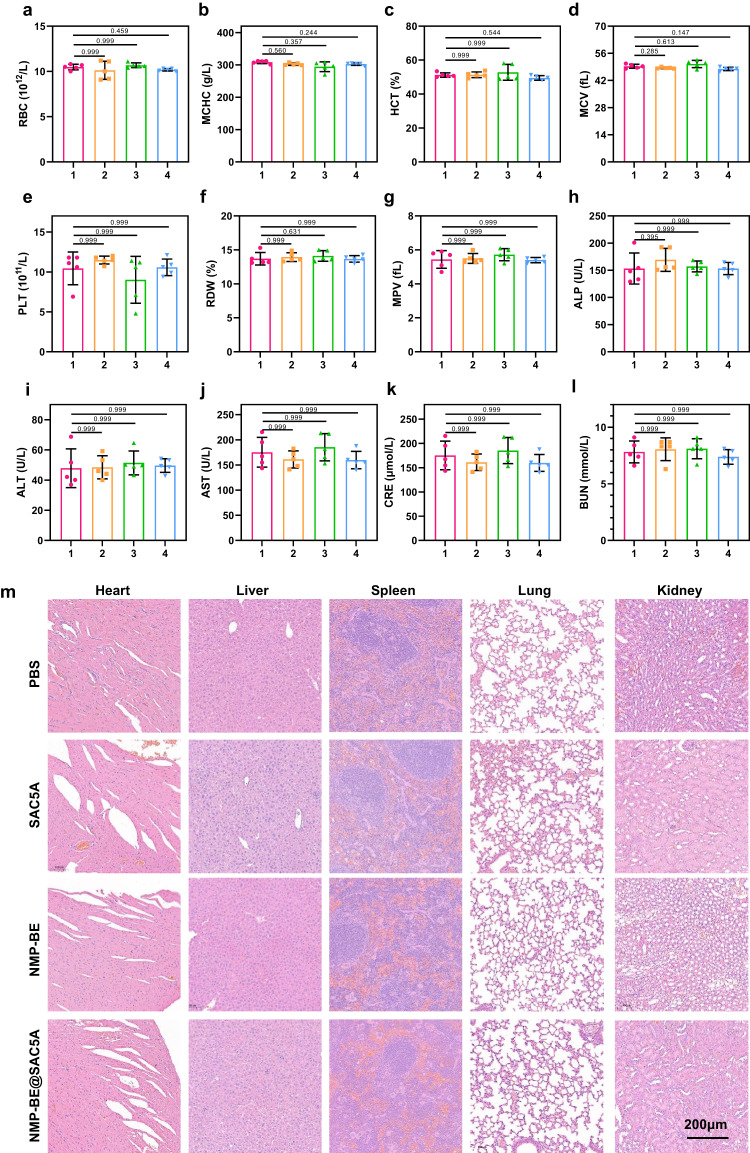
Biosafety evaluation of NMP-BE@SAC5A in vivo. RBC (**a**), MCHC (**b**), HCT (**c**), MCV (**d**), PLT (**e**), RDW (**f**), MPV (**g**), ALP (**h**), ALT (**i**), AST (**j**), CRE (**k**), and BUN (**l**) levels from the mice treated with PBS (1), SAC5A (2), NMP-BE (3), and NMP-BE@SAC5A (4) (*n* = 5 mice per group). **m** H&E stains of major organs from the mice treated with PBS, SAC5A, NMP-BE, and NMP-BE@SAC5A. Representative images from *n* = 5 mice per group. In (**a**–**l**), Data are presented as mean ± SD. *P* values are calculated by one-way ANOVA with Bonferroni’ multiple comparisons test: there are no significance versus PBS group. Source data are provided as a Source Data file.

Toxic effects are a barrier to overcome before efficient deliverability can be achieved with BE. As well as blood routine assays, blood chemistry assays were performed to evaluate the biosafety of SAC5A and NMP-BE@SAC5A. In the mice treated with SAC5A, there was a small alteration in the values of red blood cells (RBC), mean corpuscular hemoglobin concentration (MCHC), red blood cell-specific volume (HCT), mean corpuscular volume (MCV), platelets (PLT), red blood cell volume distribution width (RDW), and mean platelet volume (MPV) as shown in Fig. 6a–g. This indicated that the acute inflammation caused by SAC5A could be negligible. Moreover, no obvious increase in indicators including alkaline phosphatase (ALP), alanine transaminase (ALT), aspartate transaminase (AST), creatinine (CRE), and blood urea nitrogen (BUN), which were in close relation to liver and kidney function, was observed in the NMP-BE@SAC5A-treated group (Fig. 6h–l) demonstrating no apparent liver and kidney damage arising from NMP-BE@SAC5A. In addition, we collected the main organs (i.e., heart, liver, spleen, lung, and kidneys) in the mice treated with NMP-BE@SAC5A and performed H&E staining for histopathological examination. Accordingly, no obvious signs of inflammation or abnormality in histopathology was found, further proving that NMP-BE@SAC5A did not negatively affect major organs and may be safe for the in vivo treatment of pancreatic cancer.

## Discussion

BE is unique among a series of hypoxia-selective bioactive compounds since it exerts anticancer effects via a mechanism that is covalently binding to the Cys234 residue of eukaryotic translation factor eEF1A1. In previous work, it was demonstrated that the eEF1A1 level of xenografted tumors positively correlated with the in vivo therapeutic effects of BE^31^. The inhibition of NMP-BE and NMP-BE@SAC5A on cell proliferation is also related with the expression of eEF1A1. As overexpression and malfunction of translation protein occur commonly in tumors, the protein synthesis machinery is a potential target for anticancer therapy^70^. eEF1A1 levels are commonly higher in cancer relative to normal tissue^71^. Gozani et al. reported that post-translation modification of eEF1A is identified to promote tumorigenesis in Ras-driven cancers in vivo^72^. However, we did not uncover that NMP-BE clearly suppress tumor protein synthesis or BE inhibit the expression of eEF1A1 itself^32^. Those studies about the relationship between eEF1A1 and hypoxia, and the detailed mechanism after BE in hypoxia acting on eEF1A1 are currently underway and will be reported in another independent research^32^. In 2018, the Therapeutic Goods Administration of Australia approved plitidepsin, a naturally occurring eEF1A2 (sharing a 98% of resemblance on the level of amino acids with eEF1A1) inhibitor, for use as a first-in-class drug in the treatment of multiple myeloma^73,74^. The potential for bench-to-bedside translation of covalent cytotoxin BE acting on eEF1A1 needs further clarification.

Targeting hypoxia in tumors was regarded as a promising treatment strategy for a long time, but it was not yet implemented with widespread success and required a breakthrough, especially for compounds featuring different hypoxia-selective mechanisms.

The primary results from this study were that calixarene-aided delivery of the hydrophobic and covalent cytotoxin BE achieved significant pancreatic cancer suppression in vivo without severe acute toxicity observed. In this course, the host SAC5A was designed rationally with excellent water solubility and a deeper cavity to accommodate NMP-BE as well as imaging agents. SAC5A can rapidly respond to hypoxia to release its payloads within tumor cells, which is the first hypoxia-responsive process. The guest NMP-BE was designed as a prodrug, the mask of the active and susceptible electrophilic site may work like a cushion and alleviate the toxicity arising from payload leakage and off-target. The second hypoxia-responsive process relied on the hypoxia-sensitive feature of BE itself. When transported into hypoxic pancreatic cancer cells, BE could be finally released from NMP-BE to act on its target. The inhibitory potency of NMP-BE@SAC5A dropped against normal cells, which may benefit to overcome the side effects of BE or NMP-BE used directly. In addition, it is possible that in the encapsulated formulation which mediated by EPR effect, active components are more bioavailable for uptake by tumor cells and less available to normal cells, although further confirmatory studies toward this supposition were required.

In this work, we validated the dual hypoxia-responsive proof-of-concept^75–78^ with an example of NMP-BE@SAC5A. The in vivo data demonstrated that NMP-BE@SAC5A was a high-priority complex for more comprehensive anti-pancreatic cancer preclinical research, such as the stability, safety evaluation, metabolism and pharmacokinetics of the supramolecular formulation. Therapeutic effects of the supramolecular formulation can be further explored in more advanced and clinically relevant patient-derived xenograft models of pancreatic cancer. This is particularly true when combined with other treatment modalities, such as radiation, that preferentially eliminate the normoxic cancer cell populace. Moreover, the heterogeneity of patients’ reductase remains an important challenge for the clinical efficacy of NMP-BE@SAC5A, and we need to do more research in the future.

Overall, we presented a strategy for the modification of covalent compounds to overcome their low water solubility, efficacy and side effects. The supramolecular carrier can be conveniently replaced with appropriate groups to accommodate covalent inhibitors. Consequently, such design principle provides a direction for the efficient delivery of anticancer covalent inhibitors (i. e. BTK, KRAS, EGFR inhibitors) within other hypoxic cancers.

## Methods

### Ethics statement

All animal studies were performed in accordance with the Regulations for the Administration of Affairs Concerning Experimental Animals (Tianjin, revised in June 2018) and compliance with the Guiding Principles in the Care and Use of Animals of the American Physiological Society and approved by the Institutional Animal Care and Use Committee (IACUC) of Nankai University (Tianjin, China) (Approval number 2021-SYDWLL-00368, 2023-SYDWLL-000487). The maximal tumor size in this study models (mice) never exceeded 1.5 cm in diameter as allowed by the above ethics committee.

### Chemicals

All the reagents and solvents were commercially available and used as received unless otherwise specified purification. Sulfobutyl ether-*β*-cyclodextrin (SBE-*β*-CD) was purchased from Adamas-beta. Sodium dithionite (SDT) was purchased from J&K. Rhodamine B (RhB) and Lucigenin (LCG) were purchased from Aladdin Co., Ltd. Nile red (NR) was purchased from meilunbio Tech. Co., Ltd. 1,1’,3,3,3’,3’-Hexamethylindodicarbocyanine (CY5-DM) was obtained OKeanos Tech. Co., Ltd. Ki67 antibody was purchased from Cell Signaling Technology (9449 S, 1:1000 dilution). Adenosine triphosphate (ATP) and 4’6-diamidino-2-phenylindole (DAPI) were obtained from Solarbio. Goat anti-Rabbit IgG (H + L) Cross-Adsorbed Secondary Antibody-FITC was purchased from Thermo Fisher (F-2765, 1:1000 dilution). In situ terminal deoxynucleotidyl transferase dUTP nick end labeling (TUNEL) kits were purchased from Beyotime Biotechnology. Sulfonatocalix[5]arene (SC5A) and sulfonated azocalix[5]arene (SAC5A) were synthesized and purified according to literature procedures. Reagents for the synthesis of *N*-methyl-piperizine-BE-43547A_2_ (NMP-BE) were purchased at the highest commercial quality and used without purification. Solvents for chromatography were used as supplied by Tianjin Reagents chemical. Reactions were monitored by thin layer chromatography (TLC) carried out on silica gel plates using UV light as visualizing agent and aqueous phosphomolybdic acid or basic aqueous potassium permanganate as developing agents. 200–300 mesh silica gel purchased from Qingdao Haiyang Chemical Co., China and was used for column chromatography

### Samples

The pH 7.4 PBS solution was made by dissolving precise quantities of sodium phosphate monobasic dehydrate, disodium phosphate, sodium chloride, and potassium chloride in double-distilled water. The volume was then adjusted to 1000 mL with double-distilled water. The samples of NMP-BE@SAC5A were prepared by grinding.

### Apparatus

^1^H and ^13^C nuclear magnetic resonance (NMR) spectra were recorded on a Bruker AV400 spectrometer or a Zhongke-Niujin BIXI-I 400 spectrometer for structural characterization of compounds. Fluorescence measurements were recorded on a Cary Eclipse for the fluorescence titrations. Fluorescence microscopy images were examined using a confocal laser scanning microscope (Leica TCS SP8). Tissue sections were observed and photographed with an optical microscope (CX41, Olympus, Japan).

### In vitro release rate of BE-43547A2 (BE) from prodrug NMP-BE

NMP-BE was weighed and dissolved in HEPES (0.01 M) with pH of 7.4, then prepared into a solution with a concentration of 5 μg/mL. The buffer was extracted at different time points, and the peak areas of BE were detected by high performance liquid chromatography (HPLC), and the concentration of BE was calculated by external standard method. HPLC conditions: Shimadzu 20AT high performance liquid chromatography system; the mobile phase is 90% acetonitrile (0.1% H_3_PO_4_) and 10% water (0.1% H_3_PO_4_); the flow rate is 1 mL/min; the detector is UV detector; detection wavelength 210 nm for NMP-BE and 254 nm for BE; chromatographic column is C18 column.

### Data analyses of fluorescence titrations

Fluorescence titrations of SAC5A, SC5A^79^, and SBE-*β*-CD were performed in 10 mM PBS, pH 7.4. The complexation of SAC5A with reporter dye (RhB) was measured by direct fluorescence titrations. A mixed solution containing known amounts of SAC5A and RhB was sequentially injected into 2.50 mL RhB solution in a quartz cuvette. The dye concentrations in mixed solution and cuvette are the same to keep dye concentration constant in the course of titrations. The fluorescence intensity was measured (*λ*_ex_ = 554 nm for RhB) before the first addition and after every addition until a plateau was reached. By fitting the fluorescence intensity (*λ*_em_ = 575 nm for RhB) according to a 1:1 host-guest binding stoichiometry, the association constant was obtained. SC5A (LCG) and SBE-*β*-CD (NR) used the same method.

The complexation of SAC5A with NMP-BE was measured by competitive fluorescence titrations. A mixed solution containing known amounts of reporter dye (RhB), host (SAC5A) and competitive guest (NMP-BE) was injected into 2.50 mL RhB and SAC5A solution in a quartz cuvette. Care was taken to keep the concentrations of dye and SAC5A constant in the course of titrations. The fluorescence intensity was measured (*λ*_ex_ = 554 nm for RhB) before the first addition and after every addition. The association constant was obtained by fitting fluorescence intensity (*λ*_em_ = 575 nm for RhB) according to a 1:1 competitive binding model. The fitting of data from direct titrations and competitive titrations was performed in a nonlinear manner, and the fitting modules were downloaded from the website of Prof. Nau’s group (http://www.jacobs-university.de/ses/wnau) under the column of “Fitting Functions”.

### Cell culture

PANC1 and hTERT-HPNE cell lines were purchased from BeNa Culture Collection (Beijing, China, BNCC352264, BNCC338221), authenticated by STR profiling and tested for mycoplasma contamination. All cells were tested for mycoplasma contamination and had no mycoplasma contamination. None of the cell lines used are classified as commonly misidentified lines. Cells were cultured in DMEM medium (Corning, Manassas, VA, USA) supplemented with 10% fetal bovine serum (FBS, Gibco Life Technologies, Grand Island, NY, USA) in a humidified incubator with 5% CO_2_ at 37 °C. A humidified atmosphere containing 5% CO_2_ was used as normoxic cell culture environment. The hypoxic cell culture environment was adjusted by purging gas mixture (94% N_2_, 5% CO_2_, 1% O_2_).

### In vitro cytotoxicity assays

To determine the cell viabilities of NMP, BE, and NMP-BE, PANC1 cells (5 × 10^3^ cells/well) were seeded into the 96-well plate and incubated overnight. Then various concentrations of NMP, BE or NMP-BE were added to cells. After 72 h, 20 μL MTT solution (5 mg/mL) was added and incubated for 4 h. The OD values were determined at 570 nm by using a micro-plate reader. And the IC_50_ were calculated by GraphPad Prism. To determine the hypoxic selectivity of NMP-BE, PANC1 cells (5 × 10^3^ cells/well) were seeded into the 96-well plate. After 24 h, the cells were treated with NMP-BE of different concentrations and incubated for 24 h under normoxic or hypoxic conditions, respectively. And the cell viabilities were measured by MTT assays. To determine the cell viabilities of BE, NMP-BE, and NMP-BE@SAC5A on normal cell line, HPNE cells (5 × 10^3^ cells/well) were seeded into the 96-well plate and incubated overnight. Then various concentrations of BE, NMP-BE or NMP-BE@SAC5A were added to cells. After 24 h treatment of compounds, cell viabilities were measured by MTT assays.

The cytotoxicity assays of SAC5A and SBE-*β*-CD and the anti-proliferation ability of NMP-BE, NMP-BE@SBE-*β*-CD, and NMP-BE@SAC5A were measured by cell counting kit-8 assays. PANC1 cells (5 × 10^3^ cells/well) were seeded into the 96-well plate and incubated overnight. They were treated with SAC5A or SBE-*β*-CD of different concentrations and incubated for 24 h under normoxic or hypoxic conditions, respectively. In addition, the cells were treated with NMP-BE, NMP-BE@SBE-*β*-CD, and NMP-BE@SAC5A of different concentrations. After a 6 h incubation under normoxic conditions, the culture medium was exchanged with fresh medium. Subsequently, the cells were incubated at 37 °C for 18 h under either normoxic or hypoxic conditions, respectively. And then, the culture medium was replaced with fresh medium and 10 μL cell counting kit-8 solution (C6005, US Everbright, Jiangsu, China). Following a 4 h incubation, the optical density was assessed at 450 nm employing a microplate reader.

### Confocal laser scanning microscopy (CLSM)

PANC1 cells (1 × 10^5^ cells/well) were seeded into the confocal imaging chambers. After 24 h, the cells were treated with CY5-DM, CY5-DM@SBE-*β*-CD, and CY5-DM@SAC5A (10/10 μM). After a 6 h incubation under normoxic conditions, the culture medium was exchanged with fresh medium. Thereafter, the cells were incubated at 37 °C for 18 h under either normoxic or hypoxic conditions, respectively. Subsequently, the cells were fixed in 4% paraformaldehyde (PFA; Biosharp, Hefei, China) for 15 min, washed with PBS buffer three times and then imaged using CLSM. Cell nuclei were counterstained with DAPI (C0060, Solarbio, Beijing, China) for 5 min.

### Cellular uptake of SAC5A

To examine the cellular uptake mechanism of SAC5A complex, PANC1 cells (3 × 10^5^ cells/well) were seeded into the six-well plates and incubated overnight. The cells were pretreated with different endocytosis inhibitors for 1 h: chlorpromazine (CHP, 20 μM, an inhibitor of clathrin-mediated endocytosis), Amiloride (AMI, 500 μM, an inhibitor of macropinocytosis), Genistein (GEN, 200 μM, an inhibitor of caveolae-mediated endocytosis), methyl-*β*-cyclodextrin (M-*β*-CD, 5 mM, an inhibitor of lipid rafts-mediated endocytosis), the cells were also pre-incubated under 4 °C for 1 h (energy-dependent endocytosis). Next, the cells were treated with CY5-DM@SAC5A (10/10 μM) for another 1 h. After that, the cells were collected and washed three times with PBS, and then analyzed by flow cytometry. All experiments were carried out four times^80^.

To verify the co-localization of SAC5A and lysosome, PANC1 cells (2 × 10^4^ cells/well) were seeded into confocal dish and incubated overnight. Then the cells were treated with CY5-DM@SAC5A (10/10 μM) for 1, 3, and 6 h, respectively. After that, cells were stained with Lyso Tracker (50 nM) for 30 min at 37 °C, and then stained with DAPI. The cells were washed with PBS buffer and imaged using CLSM.

### In vivo fluorescence imaging

For animals and tumor model, male BALB/c nude mice at 5–6 weeks were purchased from Vital River Laboratory Animal Technology (Beijing, China). All animal experiments were performed according to the institutional animal care guidelines established by the Institutional Animal Care and Use Committee of Nankai University. To establish the PANC1 tumor-bearing mouse model, 1 × 10^6^ PANC1 cells were injected subcutaneously into the right chest of BALB/c nude mice. After the tumor grows to a suitable size, the tumor was divided into small pieces and then subcutaneously incubated into the right chest of other mice. When the tumor volumes of mice were around 200 mm^3^, the mice were randomized into three groups and intravenously injected with 100 μL of CY5-DM (200 μM), CY5-DM@SBE-*β*-CD (200 μM), and CY5-DM@SAC5A (200 μM). In vivo fluorescence imaging of CY5-DM, CY5-DM@SBE-*β*-CD, and CY5-DM@SAC5A were imaged by IVIS Lumina imaging system (Caliper Life Science, USA) at the time of 1, 3, 6, 12, and 24 h after injection, respectively. The ex vivo fluorescence imaging of major organs at 24 h post injection was imaged by IVIS. Fluorescent images were analyzed using Living Image 3.1 (Caliper Life Sciences). (Since cyanine dyes can be distributed in many organs due to their non-targeting nature, notably in the kidney. If the tumor was inoculated in the flank, the fluorescence signals of tumor might overlap with the signals of kidney and abdominal area during the living imaging. Therefore, to observe the distribution of the fluorescence signals overspread their bodies, the tumor was inoculated in the chest).

### Acute toxicity of NMP-BE fumaric acid

For acute toxicity of NMP-BE fumaric acid, the mice were intravenously administered with different dosage of compound (0, 10, 25, 50 mg/kg) for 10 days, respectively. Body weights were measured before administration and at daily intervals after administration (*n* = 3).

### Experimental procedure of the pharmacokinetic (PK) study of NMP-BE

All UPLC-MS/MS analysis was carried out on an Ultra performance liquid chromatographic system (ExionLC, SCIEX, USA). The chromatographic column was Waters XSelect® HSS T3 C18 (2.1 × 50 mm, 2.5 μm). Mass spectrometric detection was performed on Triple Quad^TM^ 4500 mass spectrometer with an electrospray ionization (ESI) interface operating in positive ion mode, was manufactured by SCIEX (Framingham, USA). The MS/MS system was operated at unit resolution in the multiple reaction monitoring (MRM) mode, and the monitored transitions were m/z 664.6 → 196.4 for NMP-BE, m/z 564.5 → 167.2 for BE. Male CD-1 mice at 6–8 weeks were purchased from Vital River Laboratory Animal Technology (Beijing, China). Mouse were housed at 22 ± 2 °C and 55 ± 5% (relative humidity) under a 12 h light-dark cycle. Blood samples were collected at 2, 5, 15, 30, 45 min and 1, 2, 4, 8 and 24 h post-dose into heparinized tubes. Plasma was obtained after centrifugation and stored at –80 °C until they were analyzed. The plasma concentration-time profiles of NMP-BE and BE in mouse were plotted.

### Detection of tumor hypoxia

To evaluate the tumor hypoxia at the early stage in PANC1-bearing BALB/c nude mice, immunofluorescence analysis was conducted using a Hypoxyprobe^TM^−1 Omni Kit (HP3−100; Hypoxyprobe, Burlington, MA, USA). Pimonidazole HCl (Hypoxuprobe-1, 60 mg/kg) was injected intraperitoneally 1 h before they were killed. Staining was performed according to the manufacturer’s instructions. The tissue sections were cut and fixed in cold acetone for 10 min. The sections were rinsed and incubated overnight at 4 °C with rabbit anti-pimonidazole antibodies (PAb2627AP, 1:100). The sections were then incubated for 2 h with FITC-conjugated goat-anti-rabbit antibody (Thermo, 1:1000). Between all steps of the staining procedure, the sections were rinsed three times with for 5 min in PBS and imaged by CLSM.

### In vivo anticancer efficacy study

For in vivo anticancer efficacy study, 1 × 10^6^ PANC1 cells were injected subcutaneously into the right flanks of the BALB/c nude mice. To investigate the anticancer effects of SBE-*β*-CD@NMP-BE, the mice with tumor volumes at around 50 mm^3^ were randomized into four groups (6 mice per group) and injected intravenously via tails with 200 μL of PBS, NMP-BE (5 mg/kg), SBE-*β*-CD (15 mg/kg) and NMP-BE@SBE-*β*-CD (20 mg/kg) every two days for 8 times and the tumor volumes for 14 days were continuously monitored. Tumor were measured by using a Vernier calipers and the volume (*V*) was calculated to be *V* = *d*^2^ × *D/2*, where *d* is the shortest and the *D* is longest diameter of the tumor in mm respectively. To investigate the anticancer effects of NMP-BE@SAC5A, the mice with tumor volumes at around 50 nm^3^ were randomized into four groups (6 mice per group) and injected intravenously via tails with 200 μL of PBS, NMP-BE (5 mg/kg), SAC5A (15 mg/kg), NMP-BE@SAC5A (20 mg/kg) every two days for 10 times and the tumor volumes for 14 days were continuously monitored. Tumors were measured as mentioned above. To assess potential toxicities, mice were monitored for weight loss. Tumors were collected for H&E analysis and immunofluorescence staining. Major organs including heart, liver, spleen, lung, and kidney, were collected and stained with H&E for histopathologic analysis. To evaluate biosafety of SAC5A, blood samples were collected for blood chemistry assay and blood routine assay.

### Statistical analysis

All results are presented as the mean ± standard deviation (SD) as indicated. Data were analyzed by one- or two-way analysis of variance (ANOVA) of comparison of multiple groups using the GraphPad Prism. *P* values less than 0.05 were considered statistically significant.

### Reporting summary

Further information on research design is available in the Nature Portfolio Reporting Summary linked to this article.

### Supplementary information

Supplementary Information

Reporting Summary

### Source data

Source Data

## Data Availability

The data supporting the findings of this study are available within the paper and its Supplementary Information, and from the corresponding author upon request. The source data underlying Figs. 2c–e, 3d–f, 4b, c, 5b, d, e, 6a–l and Supplementary Figs. 7, 8, 9, 10, 11, 12, 14, 15a, b, 16, 19a, b, 20, 22a, c, and 23a, c, d are provided as a Source Data file. The full image dataset is available from the corresponding author upon request. Source data are provided with this paper.
