# Supplementary material for: Dual hypoxia-responsive supramolecular complex for cancer target therapy
Source: Nat Commun. 2023 Sep 13;14:5634. doi: 10.1038/s41467-023-41388-2 (PMC10500001; doi:10.1038/s41467-023-41388-2)
Supplement: Supplementary file 1 — Supplementary Information [file 41467_2023_41388_MOESM1_ESM.pdf]

Supplementary Information

For

Dual Hypoxia-responsive Supramolecular Complex for Cancer Target Therapy

Jian-Shuang Guo,[1,4] Juan-Juan Li,[2,4] Ze-Han Wang,[2] Yang Liu,[1] Yu-Xin Yue,[2] Hua-Bin Li,[2] Xiu-He Zhao,[1] Yuan-Jun Sun,[1] Ya-Hui Ding,[3] Fei Ding,[2] Dong-Sheng Guo,[2]* Liang Wang,[3]* Yue Chen[3]

[1]College of Pharmacy, State Key Laboratory of Medicinal Chemical Biology, Tianjin Key Laboratory of Molecular Drug Research, Nankai University, Tianjin 300353, China.

[2]College of Chemistry, State Key Laboratory of Elemento-Organic Chemistry, Key Laboratory of Functional Polymer Materials (Ministry of Education), Nankai University, Tianjin 300071, China.

[3]College of Chemistry, State Key Laboratory of Medicinal Chemical Biology, Nankai University, Tianjin 300071, China.

[4]These authors contributed equally: Jian-Shuang Guo, Juan-Juan Li.

E-mail: lwang@nankai.edu.cn, dshguo@nankai.edu.cn

1 Supplementary Methods

1.1 Synthesis of SAC5A.

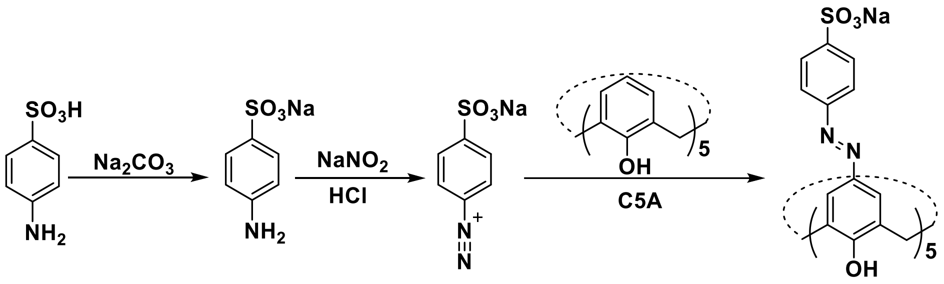

Supplementary Figure 1. The synthetic route of SAC5A.[1]

SAC5A: Sulfanilic acid (1.732 g, 10 mmol) was dissolved in water (10 mL) containing sodium carbonate (0.518 g, 5.0 mmol) at 50−55 °C. A solution of $NaNO_2$ (0.702 g, 10 mmol in 10 mL of water) was added to sulfanilic acid solution and the resulting mixture was added slowly to the concentrated HCl (4.0 mL) at 0−5 °C for 30 min and further stirred at this temperature for 1.5 h. The resultant 4-sulfobenzenediazonium chloride salt solution was slowly added into a solution of 25,26,27,28-tetrahydroxycalix[5]arene (C5A, 1.1 g, 2.0 mmol) and sodium acetate trihydrate (4.08 g, 30 mmol) in MeOH-DMF (26 mL, 5:8, v/v) to obtain a red suspension. The red mixture was allowed to stir for 2 h more in an ice bath, then acidified by 150 mL of aqueous HCl (0.25%) and warmed at 60 °C for 30 min to produce reagent as a reddish viscous solid. The residue was recrystallized with water/methanol (60 mL, 1:1, v/v), then the solution was cooled, filtered, and dried to provide a reddish solid (SAC5A) in a quantitative yield.

^{1}H NMR (400 MHz, DMSO-d_6) δ 7.81 (s, 10H, calix-Ar-H), 7.77 – 7.71 (m, 20H, Ar-H), 3.96 (s, 10H, Ar-CH_2-Ar) ppm;

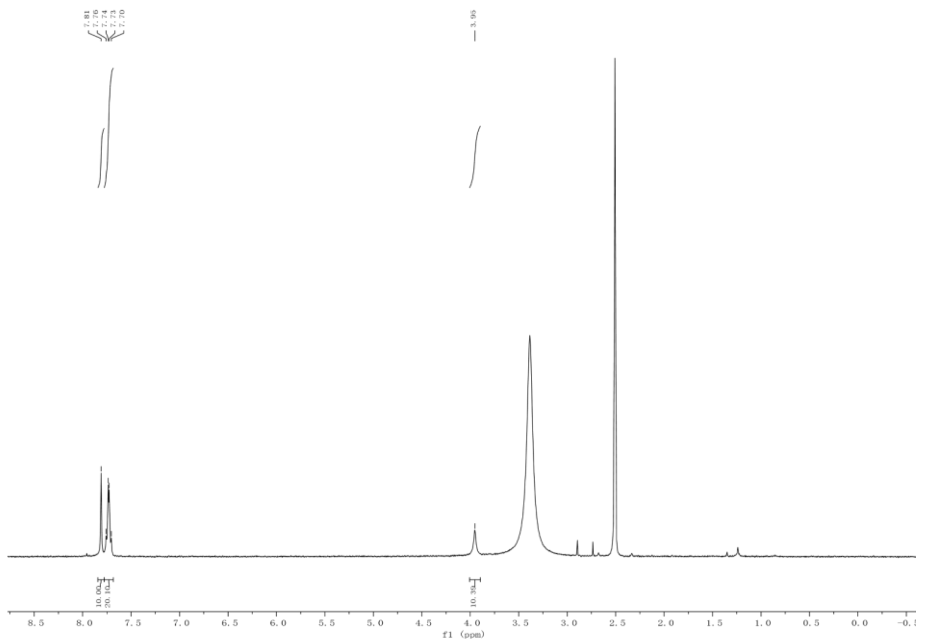

Supplementary Figure 2. ^{1}H NMR spectrum of SAC5A in DMSO-d_6, 400 MHz, 25 °C.

1.2 Synthesis of NMP-BE.

Method 1

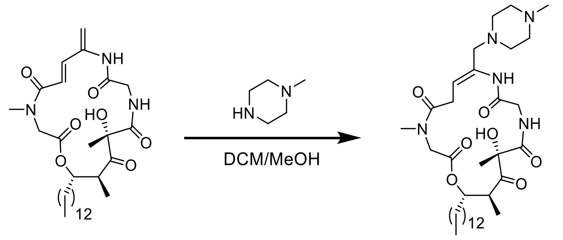

BE **NMP-BE**

Method 2

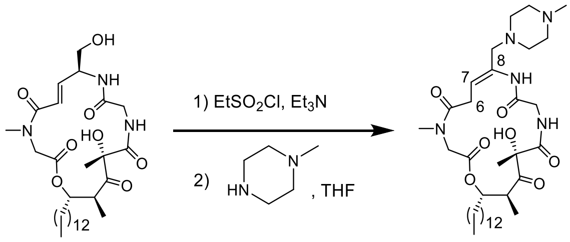

BE-OH **NMP-BE**

Supplementary Figure 3. The synthetic routes of **NMP-BE**.

NMP-BE: To a solution of BE (100 mg, 0.177 mmol, in 2 mL of DCM/MeOH (4:1)) was added with *N*-Methyl-piperazine (217 µL, 1.77 mmol). The mixture was stirred at

40 ºC for 2 h. TLC (DCM/MeOH = 10:1) showed most of BE was consumed. The resulting mixture was evaporated under reduced pressure to remove excess *N*-Methyl-piperazine. Then 5 mL of toluene was added to the residue and the mixture was evaporated under reduced pressure. The crude product was purified by silica gel chromatography (4% to 12% MeOH in DCM) to obtain a light-yellow NMP-BE (80 mg, 68%).

It was found that BE can transform into an insoluble solid during the concentration process after chromatography. To avoid/minimize product loss, we used BE-OH to generate NMP-BE as illustrated in the Supplementary Figure 3. To a solution of BE-OH (200 mg, 0.344 mmol, in 4 mL of anhydrous THF) stirred at 0 ºC was added with Et_3N (96 μL, 0.692 mmol) and $EtSO_2Cl$ (48 μL, 0.507 mmol). The mixture was stirred for around 30 min until TLC showed BE-OH was consumed. Then the reaction mixture was added with 100 μL of water to quench the excess $EtSO_2Cl$ and followed by addition of anhydrous Na_2SO_4. The mixture was filtrated, washed with EtOAc and the filtrate was concentrated under reduced pressure to obtain a residue. The residue was dissolved in 3 mL of anhydrous THF and added with 306 μL of *N*-Methyl-piperazine. The resulting mixture was heated at 40 ºC for 2 h (note: Extending the reaction time to 48 h resulted the mess of the reaction according to ^{1}H-NMR and we speculated a product with C6-C7 double bond was formed via double bond migration) until most of the penultimate ethyl sulfonyl ester was consumed according to TLC analysis. Then the mixture was directly subjected to preparative TLC (DCM/MeOH (5:1)). The product attached in silica-gel powder was eluted with (DCM/MeOH (12:1)). The eluent was evaporated under reduced pressure to obtain ≈ 5.0 mL of solution. 2 mL of *t*-BuOH and 2.0 mL of H_2O was added to the solution and the mixture was evaporated carefully. The resultant solution was lyophilized at –70 ºC to obtain NMP-BE (105 mg, 46% for 2 steps) as a freeze-dried white powder.

$[\alpha]^{14}_D$ = + 70.3 (*c* = 0.5, $CHCl_3$: MeOH = 1:1);

ν_{max} (KBr): 3430, 2959, 2924, 2854, 1750, 1659, 1520, 1460, 1402, 1261, 1194, 1054, 1033, 800, 772 cm^{-1};

^{1}H NMR (400 MHz, DMSO) δ 8.48 (t, J = 6.0 Hz, 1H), 8.37 (s, 1H), 6.57 (s, 1H), 5.50 (t, J = 6.0 Hz, 1H), 5.16 (ddd, J = 9.9, 7.2, 2.8 Hz, 1H), 4.27 (d, J = 18.9 Hz, 1H), 4.03 (d, J = 18.9 Hz, 1H), 3.88 (dd, J = 15.9, 6.8 Hz, 1H), 3.58 (dd, J = 15.9, 5.2 Hz, 1H), 3.39 (m, 1H), 3.11 (d, J = 13.7 Hz, 1H), 2.97 – 2.86 (m, 2H), 2.80 (s, 3H), 2.74 (dd, J = 18.9, 5.7 Hz, 1H), 2.35 (s, 8H), 2.18 (s, 3H), 1.57 (s, 2H), 1.49 (s, 3H), 1.23 (br s, 22H), 1.02 (d, J = 6.8 Hz, 3H), 0.85 (t, J = 6.7 Hz, 3H) ppm;

^{13}C NMR (101 MHz, DMSO) δ 209.32, 172.68, 171.03, 168.90, 166.56, 131.88, 117.90, 80.03, 75.40, 61.38, 54.51, 52.08, 50.57, 45.45, 44.15, 43.08, 34.53, 31.24, 30.77, 29.95, 28.98, 28.95, 28.90, 28.86, 28.75, 28.64, 24.65, 22.03, 21.32, 13.89, 13.67;

HRMS (ESI) *m/z*: calcd for $C_{35}H_{62}N_5O_7^+$ [M + H]$^+$: 664.4644, found: 664.4637.

a

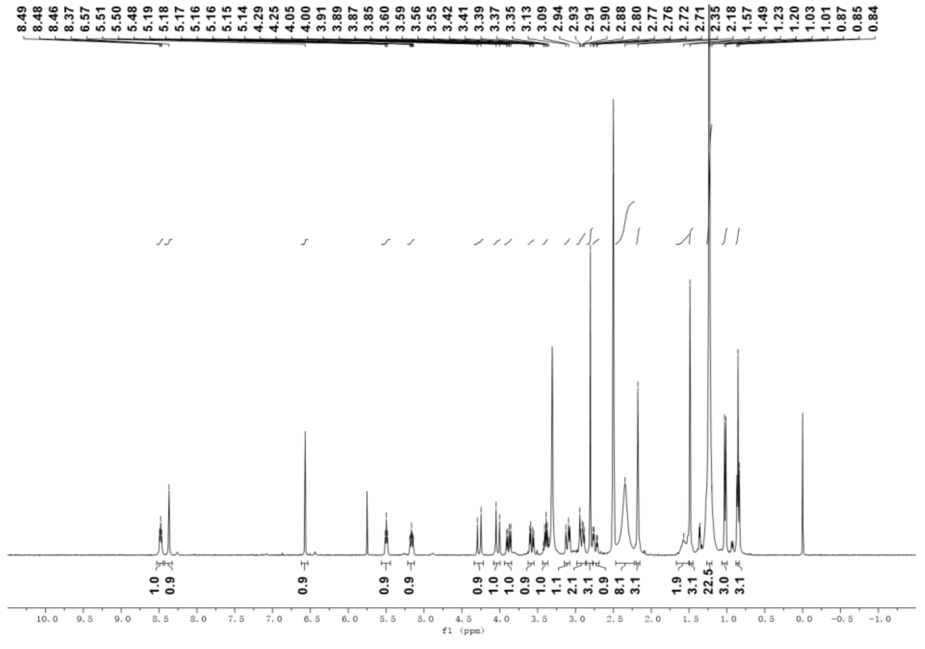

b

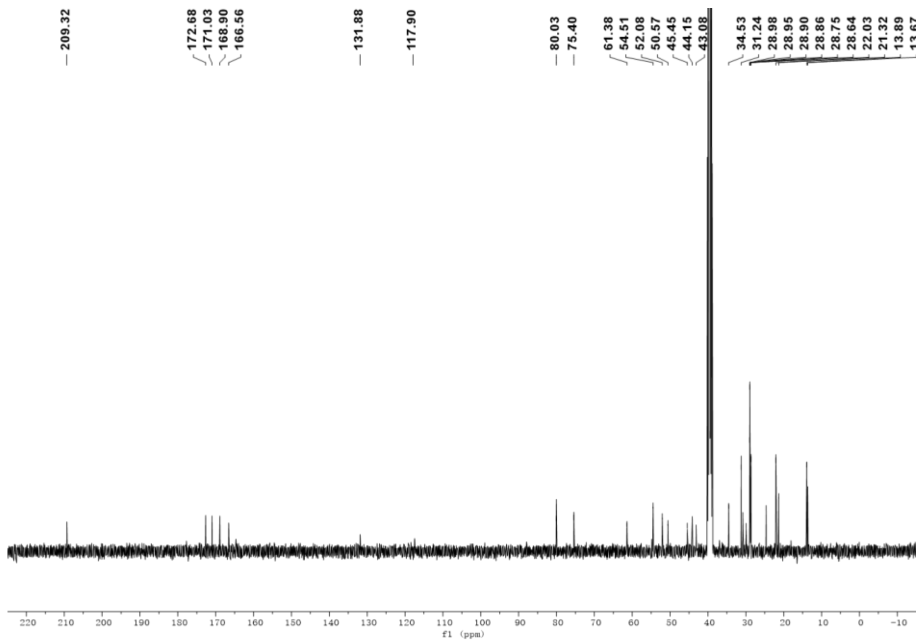

Supplementary Figure 4. (a) ^{1}H NMR spectrum of NMP-BE in DMSO-d_6, 400 MHz, 25 °C. (b) ^{13}C NMR spectrum of NMP-BE in DMSO-d_6, 101 MHz, 25 °C.

The product obtained by method 2 was further subjected to HPLC analysis, the result indicated ≈ 92.9% purity. The instrument and condition for the HPLC analysis is listed below:

Instrument: SHIMADZU 20AT

Column: Sciences Inertsil ODS-SP 3 μm, 4.6*250 mm

Detection wavelength: 210 nM

Velocity of flow: 1 mL/min

Diluent: 70% CH_3CN

Mobile phase A: CH_3CN: H_2O: H_3PO_4 = 10: 90: 0.1.

Mobile phase B: CH_3CN: H_2O: H_3PO_4 = 90: 10: 0.1.

<Chromatogram>

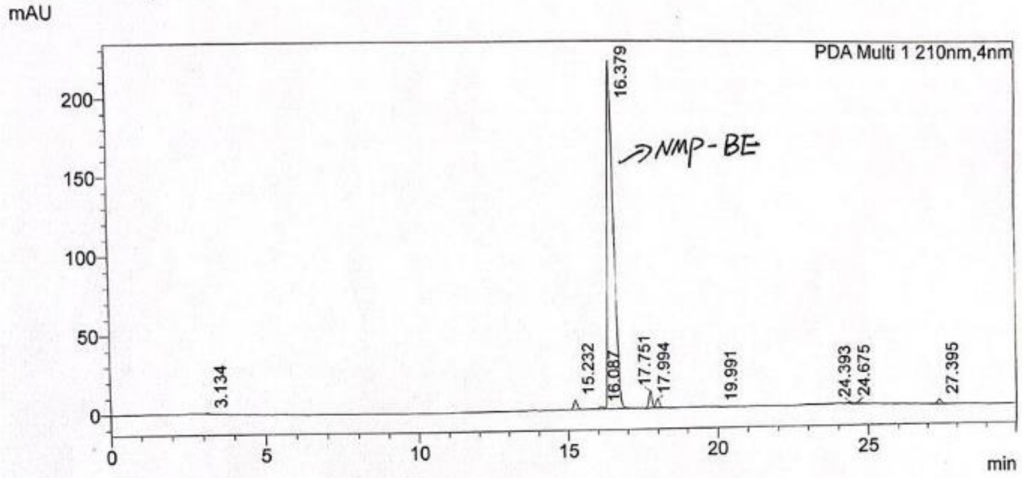

<Peak Table>

PDA Ch1 210nm

Peak#	Ret. Time	Area	Height	Conc.	Unit	Mark	Name
1	3.134	6793	807	0.194	mg/L		RT:3.229
2	15.232	49388	6313	1.407			
3	16.087	13503	1289	0.385			
4	16.379	3260699	221037	92.922		V	
5	17.751	80070	11100	2.282			
6	17.994	45142	5913	1.286		V	
7	19.991	6139	470	0.175			
8	24.393	8401	956	0.239			
9	24.675	11555	1672	0.329			
10	27.395	27386	3084	0.780			
Total		3509077	252641				

Supplementary Figure 5. The HPLC trace of NMP-BE obtained via method 2.

NMP-BE could be further purified to 98.9% purity employing preparative liquid chromatography involving 2 steps. Firstly, the sample was sujected to preparative liquid chromatography. The instrument and condition are listed below:

Instrument: Agela FL-H050G

Column: GEMINI C18 250 x 21.2 mm, 5 μm 110A

Mobile phase A: CH_3CN: H_2O: TFA = 10: 90: 0.05.

Mobile phase B: CH_3CN: H_2O = 90: 10.

Then the eluent was concentrated and lyophilized to obtain a white powder, which was subjected to the second time preparative liquid chromatography.

Instrument: Agela FL-H050G

Column: GEMINI C18 250 x 21.2 mm, 5 μm 110A

Mobile phase A: 10 mM NH_4HCO_3 (with ammonia to adjust pH to 9).

Mobile phase B: CH_3CN: H_2O = 90: 10.

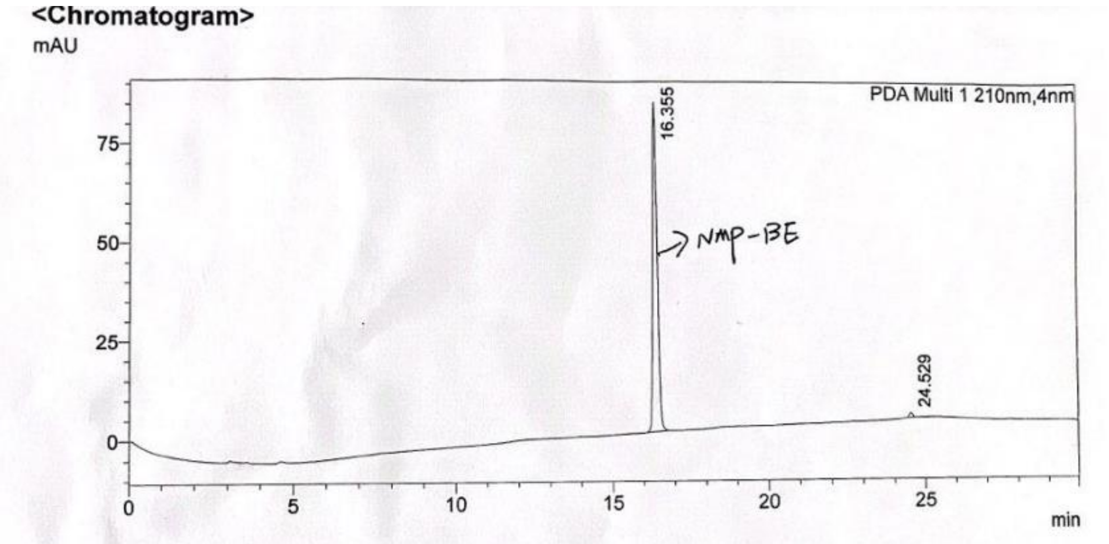

<Peak Table>

PDA Ch1 210nm

Peak#	Ret. Time	Area	Height	Conc.	Unit	Mark	Name
1	16.355	837143	83980	98.996			
2	24.529	8492	1177	1.004			
Total		845635	85157				

Supplementary Figure 6. The HPLC trace of NMP-BE after purification with preparative liquid chromatography.

Then the eluent was concentrated and lyophilized to obtain a white powder to store at −20 ºC.

2 Supplementary Notes

2.1 *In vitro* release rate of BE from prodrug NMP-BE

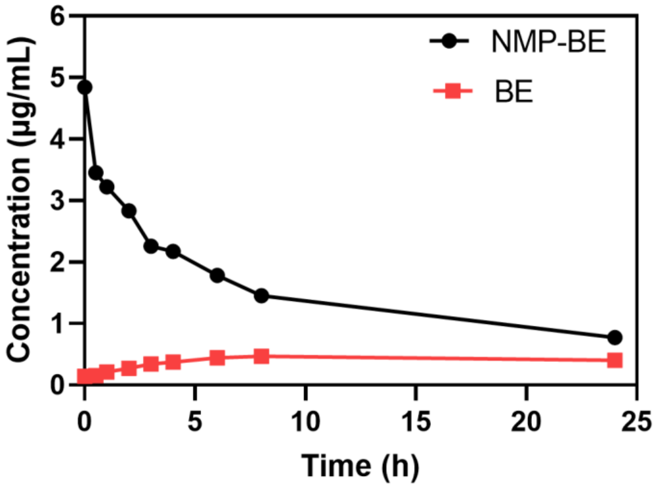

Supplementary Figure 7. *In vitro* release rate of BE. NMP-BE was prepared into a solution of HEPES (10 mM, pH = 7.4) at a concentration of 5 μg/mL. The buffer was extracted at different time points, and the peak area of NMP-BE or BE was detected by HPLC, and the concentration of NMP-BE or BE was calculated by external standard method.

We speculate that the possible reason for the low concentration of released BE is mainly the low water solubility of BE, and BE was precipitated in HEPES buffer.

2.2 Cell viability and IC_{50} of NMP, BE or NMP-BE against PANC1 cells via MTT assays

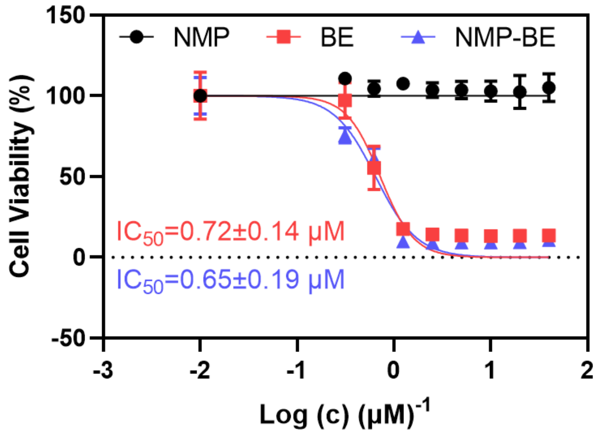

Supplementary Figure 8. Cell viabilities of PANC1 cells treated with various concentrations of NMP, BE or NMP-BE under normoxic conditions for 72 h (n = 6 biologically independent samples). IC_{50} for BE = 0.72 ± 0.14 μM; IC_{50} for NMP-BE = 0.65 ± 0.19 μM. Data are presented as mean ± SD.

2.3 Hypoxia-selective cytotoxicity of NMP-BE in PANC1 cells

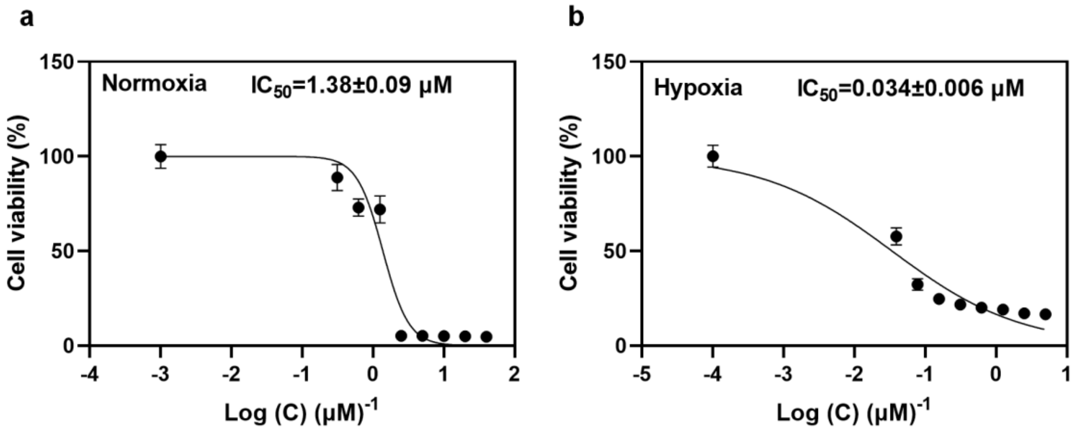

Supplementary Figure 9. Cell viabilities of PANC1 cells treated with various concentrations of NMP-BE under normoxic (a) or hypoxic (b) conditions for 24 h (n = 6 biologically independent samples). IC_{50} for NMP-BE (Normoxia) = 1.38 ± 0.09 μM; IC_{50} for NMP-BE (Hypoxia) = 0.034 ± 0.006 μM.

2.4 Acute toxicity of NMP-BE derivative in mice

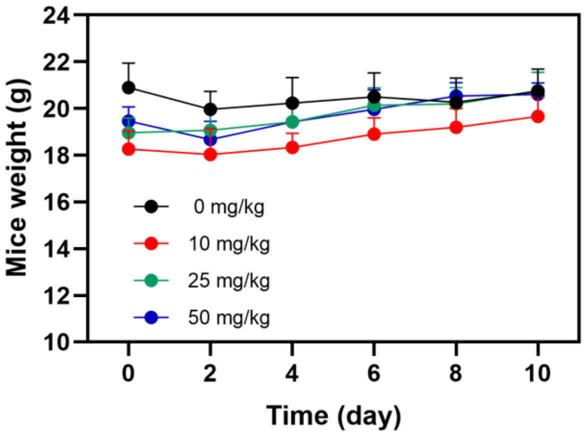

Supplementary Figure 10. Weight curves of mice administrated with different concentrations (0, 10, 25, 50 mg/kg) of NMP-BE fumaric acid on the tail vein for 10

days. Data are presented as the mean ± SD (n = 3 mice per group).

2.5 Pharmacokinetic analysis of NMP-BE

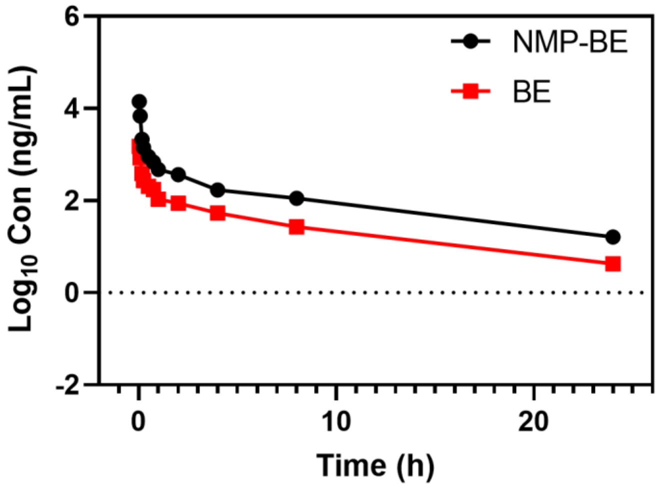

Supplementary Figure 11. PK study of NMP-BE (iv administration). BE and NMP-BE plasma concentration-time curve in mice after a single iv administration of NMP-BE (10 mg/kg). Data are presented as the mean ± SD (n = 3 mice per time point).

2.6 Job's plot for the complexation of RhB with SAC5A

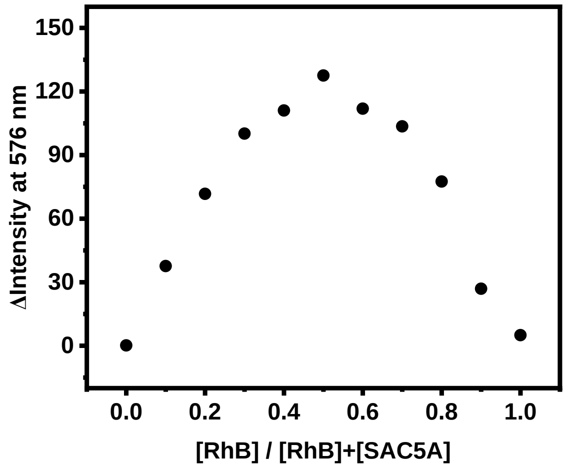

Supplementary Figure 12. Job's plot for solutions of RhB and SAC5A, λ_{ex} = 497 nm, λ_{em} = 594 nm, [RhB] + [SAC5A] = 1.0 μM.

2.7 The NOESY of NMP-BE@SAC5A

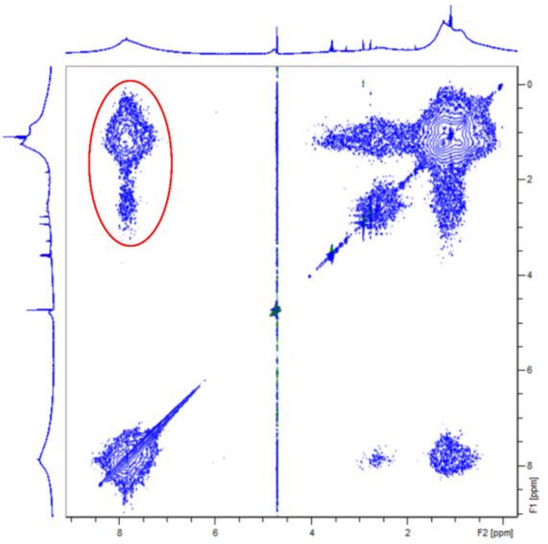

Supplementary Figure 13. The NOESY spectrum of NMP-BE@SAC5A in D_2O, 400 MHz, 25 °C. It shows a cross-peak between the alkyl chain hydrogens on NMP-BE and the phenyl hydrogens on SAC5A, thus clearly indicating the host-guest interactions between NMP-BE and SAC5A (red circle). D_2O passivates the associated hydrogen and broadens the peaks, so that the hydrogen correlation between molecules is a little scattered.

2.8 Fluorescence titrations of SC5A and SBE-*β*-CD with NMP-BE

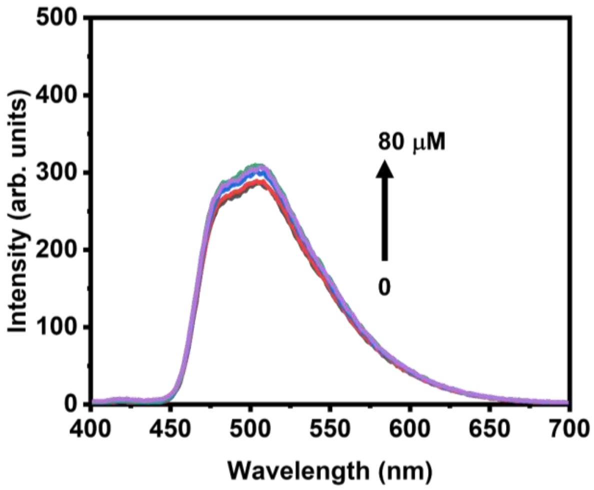

Supplementary Figure 14. Competitive fluorescence titration of LCG@SC5A (1.0/1.0 μM) with NMP-BE (up to 80 μM) in HEPES buffer (10 mM, pH = 7.4) at 25 °C, λ_{ex} =

368 nm. The 1:1 binding affinity between SC5A and LCG has been reported as $1.48 \times 10^6\ M^{-1}$ in the previous work.[2]

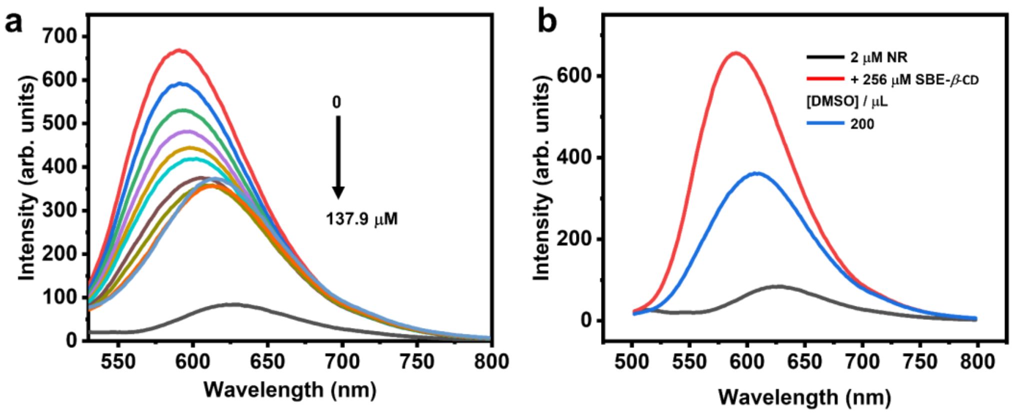

Supplementary Figure 15. (a) Competitive fluorescence titration of NR@SBE-β-CD (2.0/256 μM) with NMP-BE (up to 137.9 μM) in HEPES buffer (10 mM, pH = 7.4) at 25 °C, λ_{ex} = 500 nm. (b) The effect of DMSO (the solvent for NMP-BE) on the host-guest pair.

2.9 The cellular uptake of CY5-DM@SAC5A

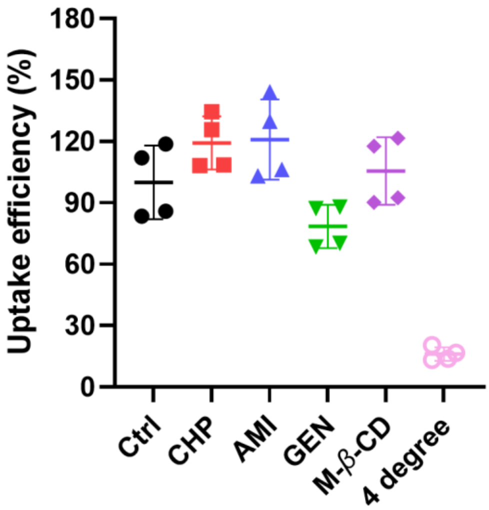

Supplementary Figure 16. Inhibition of CY5-DM@SAC5A uptake was studied using specific endocytosis inhibitors. Chlorpromazine (CHP): inhibitor of clathrin-mediated endocytosis; amiloride (AMI): inhibitor of macropinocytosis; Genistein (GEN): inhibitor of caveolae-mediated endocytosis; Methyl-β-cyclodextrin (M-β-CD): lipid rafts-mediated endocytosis; 4 °C: energy-mediated endocytosis. Data are presented as

mean ± SD (n = 4 biologically independent samples).

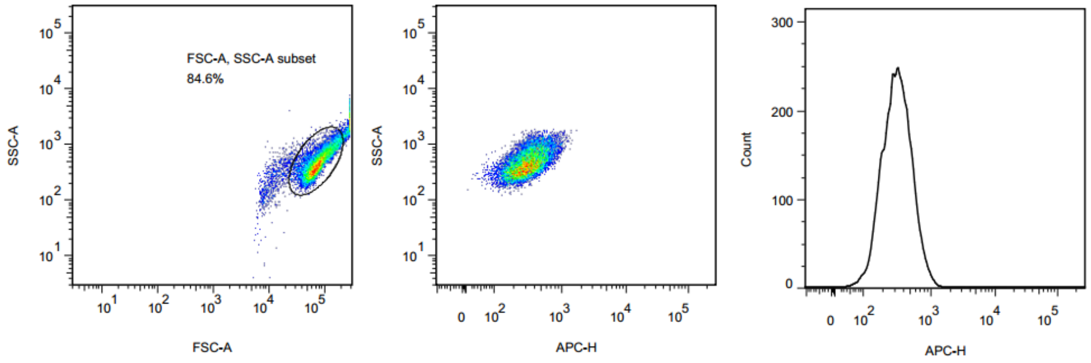

Supplementary Figure 17. Gating strategy for identification of the fluorescence of PANC1 cells treated with CY5-DM@SAC5A.

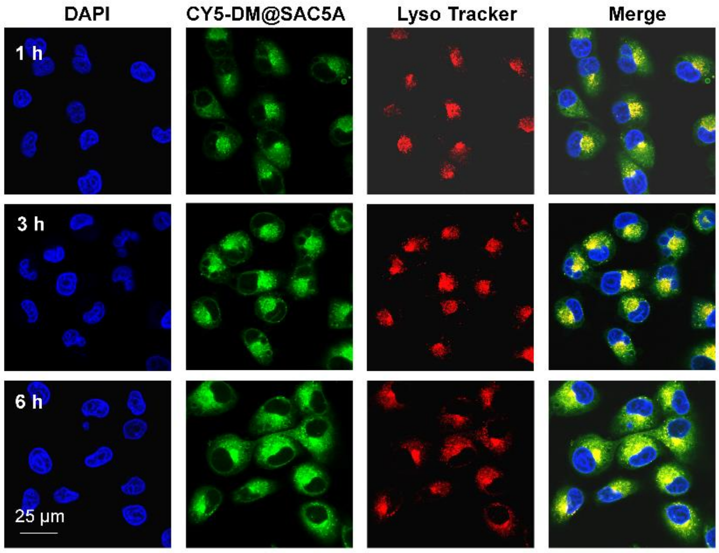

Supplementary Figure 18. Time-dependent cellular uptake of CY5-DM@SAC5A. PANC1 cells were treated with CY5-DM@SAC5A for 1, 3, and 6 h. and then *in vivo* co-localized with Lyso Tracker Red and imaged by CLSM. Blue: DAPI; Green: CY5-DM; Red: Lyso Tracker. Scale bar, 25 μm. This was repeated independently for 3 times with similar results.

2.10 *In vitro* hypoxia imaging and cytotoxicity of SBE-*β*-CD

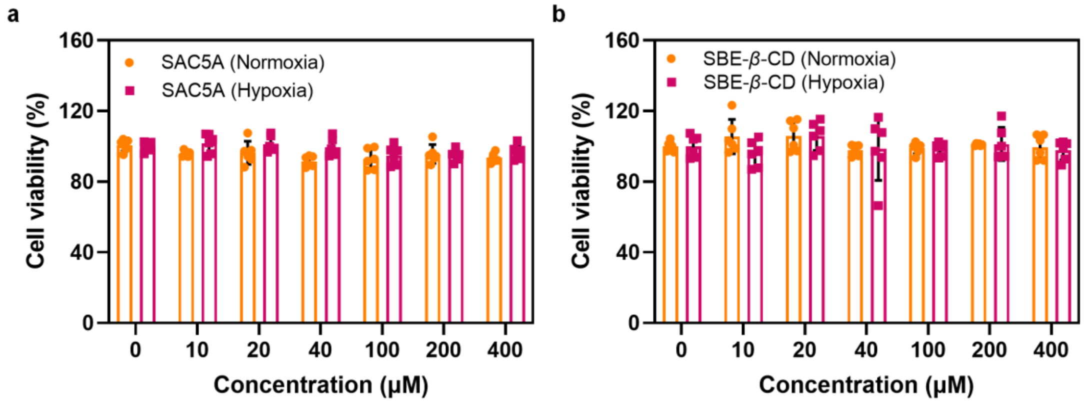

Supplementary Figure 19. The cytotoxicity of SAC5A and SBE-β-CD. (a) Cell viability of PANC1 cells treated with various concentrations of SAC5A for 24 h. (b) Cell viability of PANC1 cells treated with various concentrations of SBE-β-CD for 24 h. Data are presented as mean ± SD (n = 6 biologically independent samples).

2.11 *In vitro* cytotoxicity of BE, NMP-BE or NMP-BE@SAC5A in HPNE cells

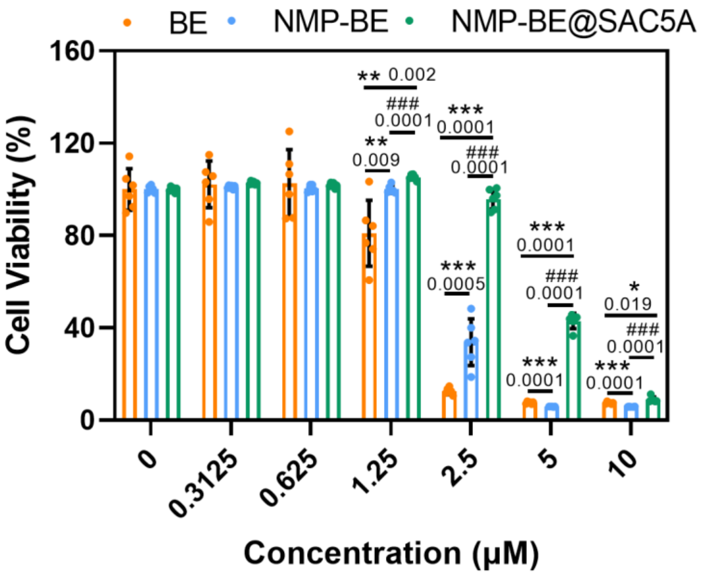

Supplementary Figure 20. Cell viability of HPNE cells treated with various concentrations of BE, NMP-BE or NMP-BE@SAC5A for 24 h. Data are presented as mean ± SD (n = 6 biologically independent samples). P values are calculated by paired t test: $^{*}P < 0.05$, $^{**}P < 0.01$, $^{***}P < 0.001$ versus BE group; $^{\#\#\#}P < 0.001$ versus NMP-BE group.

2.12 *Ex vivo* fluorescence imaging of CY5-DM@SAC5A

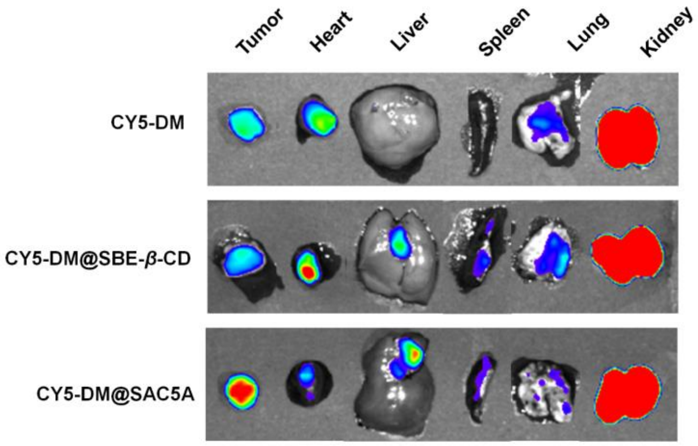

Supplementary Figure 21. The fluorescence imaging of the major organs *in ex vivo* at 24 h post injection of CY5-DM, CY5-DM@SBE-*β*-CD and CY5-DM@SAC5A.

2.13 *In vivo* anticancer effects of NMP and SAC5A

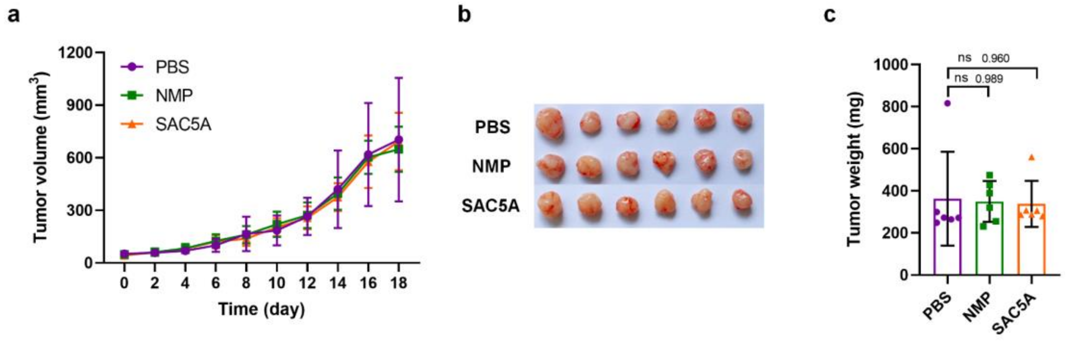

Supplementary Figure 22. In *vivo* anticancer effects of NMP and SAC5A. (a) Average tumor volume growth curves of mice treated with PBS, NMP, and SAC5A. Data are presented as mean ± SD (n = 6 mice per group). P values are calculated by two-way ANOVA with Turkey's multiple comparisons test: ns: no significance; (b) Images of dissected tumors in various groups. (c) Weights of tumors from the mice after treatment. Data are presented as mean ± SD (n = 6 biologically independent samples). P values are calculated by one-way ANOVA with Turkey's multiple comparisons test: ns: no significance.

Note: (b) During the experiment, the tumor images were taken of PBS vs NMP, PBS vs SAC5A groups respectively. As the PBS group consisted of the same tumors, the tumor

images of all three groups were ultimately combined.

2.14 *In vivo* anticancer effects of NMP-BE@SBE-β-CD

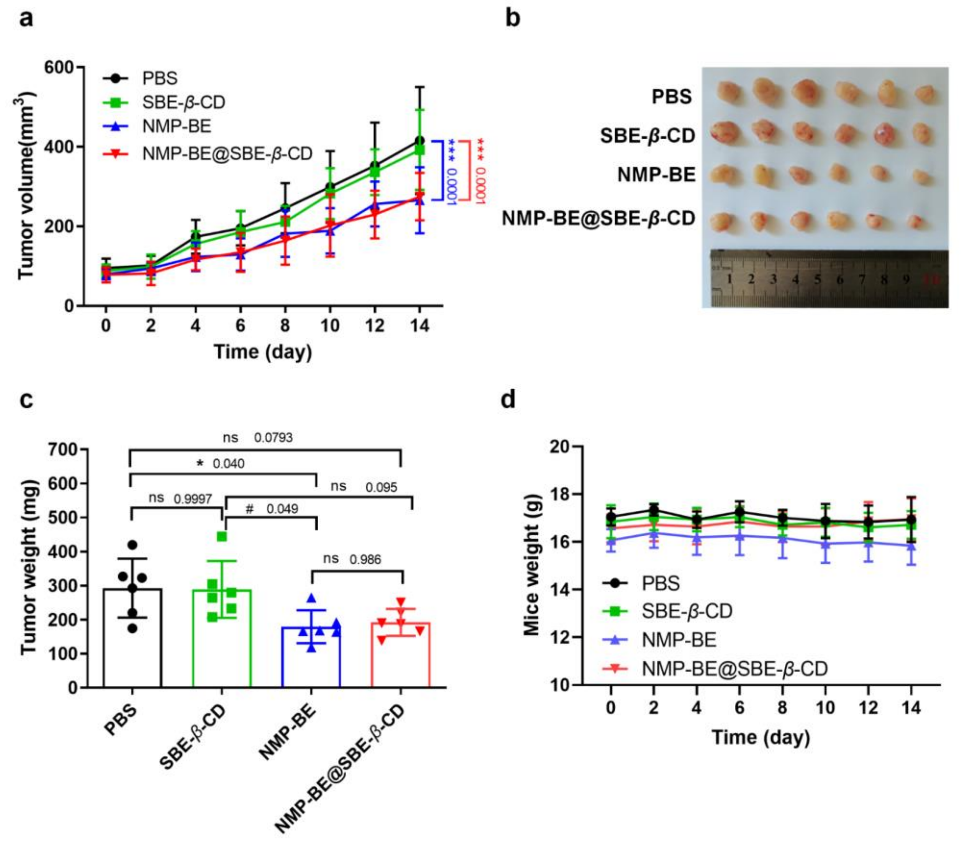

Supplementary Figure 23. In *vivo* anticancer effects of NMP-BE@SBE-β-CD. (a) Average tumor volume growth curves of mice treated with PBS, SBE-β-CD, NMP-BE, and NMP-BE@SBE-β-CD. Data are presented as mean ± SD (n = 6 mice per group). P values are calculated by two-way ANOVA Turkey's multiple comparisons test: $^{***}P$ < 0.001 versus PBS group; (b) Images of dissected tumors in various groups. (c) Weights of tumors from the mice after treatment. Data are presented as mean ± SD (n = 6 biologically independent samples). P values are calculated by one-way ANOVA Turkey's multiple comparisons test: $^{*}P < 0.05$ versus PBS group; $^{\#}P < 0.05$ versus SBE-β-CD group; ns: no significance. (d) Weight changes of the mice in different groups (PBS, SBE-β-CD, NMP-BE and NMP-BE@SBE-β-CD). Data are presented as mean ± SD (n = 6 mice per group).

Supplementary References

1. Yue, Y. X., et al. Promoting tumor accumulation of anticancer drugs by hierarchical carrying of exogenous and endogenous vehicles. *Small Struct.* 2200067 (2022).
2. Yu, H., Geng, W. C., Zheng, Z., Gao J. Guo, D. S. & Wang, Y. Facile fluorescence monitoring of gut microbial metabolite trimethylamine *N*-oxide via molecular recognition of guanidinium-modified calixarene. *Theranostics* **9**, 4624–4632 (2019).
