# Supplementary material for: Dual hypoxia-responsive supramolecular complex for cancer target therapy
Source: Nat Commun. 2023 Sep 13;14:5634. doi: 10.1038/s41467-023-41388-2 (PMC10500001; doi:10.1038/s41467-023-41388-2)
Supplement: Supplementary file 2 — Reporting Summary [file 41467_2023_41388_MOESM2_ESM.pdf]

Corresponding author(s): Dong-Sheng Guo
Last updated by author(s): August 31, 2023

Reporting Summary

Nature Portfolio wishes to improve the reproducibility of the work that we publish. This form provides structure for consistency and transparency in reporting. For further information on Nature Portfolio policies, see our Editorial Policies and the Editorial Policy Checklist.

Statistics

For all statistical analyses, confirm that the following items are present in the figure legend, table legend, main text, or Methods section.

n/a	Confirmed	
[ ]	[x]	The exact sample size (*n*) for each experimental group/condition, given as a discrete number and unit of measurement
[ ]	[x]	A statement on whether measurements were taken from distinct samples or whether the same sample was measured repeatedly
[ ]	[x]	The statistical test(s) used AND whether they are one- or two-sided<br>*Only common tests should be described solely by name; describe more complex techniques in the Methods section.*
[ ]	[x]	A description of all covariates tested
[ ]	[x]	A description of any assumptions or corrections, such as tests of normality and adjustment for multiple comparisons
[ ]	[x]	A full description of the statistical parameters including central tendency (e.g. means) or other basic estimates (e.g. regression coefficient) AND variation (e.g. standard deviation) or associated estimates of uncertainty (e.g. confidence intervals)
[ ]	[x]	For null hypothesis testing, the test statistic (e.g. *F*, *t*, *r*) with confidence intervals, effect sizes, degrees of freedom and *P* value noted<br>*Give P values as exact values whenever suitable.*
[x]	[ ]	For Bayesian analysis, information on the choice of priors and Markov chain Monte Carlo settings
[x]	[ ]	For hierarchical and complex designs, identification of the appropriate level for tests and full reporting of outcomes
[x]	[ ]	Estimates of effect sizes (e.g. Cohen's *d*, Pearson's *r*), indicating how they were calculated

Our web collection on statistics for biologists contains articles on many of the points above.

Software and code

Policy information about availability of computer code

Data collection	Photographs of H&E staining and Immunofluorescence (IF) images were collected with 3DHISTECH (P250 FLASH); Photographs of cell IF staining were collected with Leica (Leica TCS SP8). Photographs of in vivo fluorescence images were collected with IVIS Spectrum (PerkinElmer)
Data analysis	The data were analysed by using Image J 180, Living image 4.2 and Slideviewer 2.5.

For manuscripts utilizing custom algorithms or software that are central to the research but not yet described in published literature, software must be made available to editors and reviewers. We strongly encourage code deposition in a community repository (e.g. GitHub). See the Nature Portfolio guidelines for submitting code & software for further information.

Data

Policy information about availability of data

All manuscripts must include a data availability statement. This statement should provide the following information, where applicable:

- Accession codes, unique identifiers, or web links for publicly available datasets
- A description of any restrictions on data availability
- For clinical datasets or third party data, please ensure that the statement adheres to our policy

The data supporting the findings of this study are available within the paper and its Supplementary Information, and from the corresponding author upon request. Source data are provided with this paper. The source data underlying Figs. 2c, d, e, 3d-f, 4b, c, 5b, d, e, 6a-l and Supplementary Figs. 7, 8, 9, 10, 11, 12, 14, 15a, b,

16, 19a, b, 20, 22a, c, and 23a, c, d are provided as a Source Data file. The full image dataset is available from the corresponding author upon request.

Human research participants

Policy information about studies involving human research participants and Sex and Gender in Research.

Reporting on sex and gender	Not applicable
Population characteristics	Not applicable
Recruitment	Not applicable
Ethics oversight	Not applicable

Note that full information on the approval of the study protocol must also be provided in the manuscript.

Field-specific reporting

Please select the one below that is the best fit for your research. If you are not sure, read the appropriate sections before making your selection.

- [x] Life sciences
- [ ] Behavioural & social sciences
- [ ] Ecological, evolutionary & environmental sciences

For a reference copy of the document with all sections, see nature.com/documents/nr-reporting-summary-flat.pdf

Life sciences study design

All studies must disclose on these points even when the disclosure is negative.

Sample size	No statistical methods was used to predetermine the samples size. Each finding was confirmed with minimum necessary number such as 3-6 replicates for each experiments. The sample was randomly choose to be determined in each group. These sample sizes are sufficient which have reached statistical significance.
Data exclusions	No data was excluded from the analyses.
Replication	All experiments were performed with at least three technical replicates on more than one occasion to ensure reproducibility across experiments. The reproducibility was high level.
Randomization	The samples were randomly grouped.
Blinding	The experimentator was blinded while performing the ex-vivo analysis of the mice. However, blinding was not performed while performing in vivo interventions. This was done to ensure that the correct intervention was applied to each group as allocated. This was also important to ensure animal ethical guidelines and scoring based on the interventions.<br>For experiments other than those involving mice work, the experimentator was blinded to group allocation during data collection and analysis.

Reporting for specific materials, systems and methods

We require information from authors about some types of materials, experimental systems and methods used in many studies. Here, indicate whether each material, system or method listed is relevant to your study. If you are not sure if a list item applies to your research, read the appropriate section before selecting a response.

Materials & experimental systems

n/a	Involved in the study	
[ ]	[x]	Antibodies
[ ]	[x]	Eukaryotic cell lines
[x]	[ ]	Palaeontology and archaeology
[ ]	[x]	Animals and other organisms
[x]	[ ]	Clinical data
[x]	[ ]	Dual use research of concern

Methods

n/a	Involved in the study	
[x]	[ ]	ChIP-seq
[ ]	[x]	Flow cytometry
[x]	[ ]	MRI-based neuroimaging

Antibodies

Antibodies used	Mouse anti-Ki67 (CST, 9449S)

	Goat anti-Rabbit IgG (H+L) Cross-Adsorbed Secondary Antibody-FITC (Thermo, F-2765)<br>Affinity purified rabbit anti-pimonidazole (Hypoxyprobe, PAb2627AP)
Validation	All the antibodies used are from commercial sources and have been validated by the vendors. Validation data are available on the manufacturer's website.<br>1.Mouse anti-Ki67 (CST, 9449S)<br>https://www.cellsignal.cn/products/primary-antibodies/ki-67-8d5-mouse-mab/9449?site-search-type=Products&N=4294956287&Ntt=ki67&fromPage=plp<br>2.Goat anti-Rabbit IgG (H+L) Cross-Adsorbed Secondary Antibody-FITC (Thermo, F-2765)<br>https://www.thermofisher.cn/cn/zh/antibody/product/Goat-anti-Rabbit-IgG-H-L-Cross-Adsorbed-Secondary-Antibody-Polyclonal/F-2765<br>3.Affinity purified rabbit anti-pimonidazole (Hypoxyprobe, PAb2627AP)<br>http://www.hypoxyprobe.com/hp3-100kit.html

Eukaryotic cell lines

Policy information about cell lines and Sex and Gender in Research

Cell line source(s)	PANC1 and hTERT-HPNE (HPNE) cell lines were purchased from BeNa Culture Collection (Beijing, China, BNCC352264, BNCC338221)
Authentication	PANC1 and HPNE cells authenticated by STR profiling.
Mycoplasma contamination	All cells were tested to be free mycoplasma contamination.
Commonly misidentified lines<br>(See ICLAC register)	None of the cell lines used are classified as commonly misidentified lines.

Animals and other research organisms

Policy information about studies involving animals; ARRIVE guidelines recommended for reporting animal research, and Sex and Gender in Research

Laboratory animals	Male BALB/c nude mice at 5-6 weeks were applied to establish this animal model from Vital River Laboratory Animal Technology (Beijing, China). Male CD-1 mice at 6-8 weeks were applied for PK study from Vital River Laboratory Animal Technology (Beijing, China).
Wild animals	No wild animals were used in this study.
Reporting on sex	This study applied to both females and males.
Field-collected samples	The study did not involve samples collected from the field.
Ethics oversight	All animal studies were performed in accordance with the Regulations for the Administration of Affairs Concerning Experimental Animals (Tianjin, revised in June 2018) and compliance with the Guiding Principles in the Care and Use of Animals of the American Physiological Society and approved by the Institutional Animal Care and Use Committee (IACUC) of Nankai University (Tianjin, China) (Approval number 2021-SYDWLL-00368, 2023-SYDWLL-000487).

Note that full information on the approval of the study protocol must also be provided in the manuscript.

Flow Cytometry

Plots

Confirm that:

- [ ] The axis labels state the marker and fluorochrome used (e.g. CD4-FITC).
- [ ] The axis scales are clearly visible. Include numbers along axes only for bottom left plot of group (a 'group' is an analysis of identical markers).
- [ ] All plots are contour plots with outliers or pseudocolor plots.
- [ ] A numerical value for number of cells or percentage (with statistics) is provided.

Methodology

Sample preparation	PANC1 cells were seeded in 6-well plates and incubated in DMEM complete medium for 24 h. The cells were pre-treated with endocytosis inhibitors for 1 h,. Next, the cells were treated with CY5-DM@SAC5A for another 1 h. After that, the cells were collected and washed three times with PBS, and then analyzed by flow cytometry.
Instrument	BD LSRFortessa (BD Biosciences)

Software	The flow cytometry data was collected by BD FACSDiva Software (BD LSRFortessa) and analyzed by FlowJo 7.6.
Cell population abundance	We used cell lines to evaluate the cellular uptake of CY5-DM@SAC5A via detecting the CY5-DM mean fluorescence intensity.
Gating strategy	No cell exclusion was needed to determine the CY5-DM mean fluorescence intensity.

☒ Tick this box to confirm that a figure exemplifying the gating strategy is provided in the Supplementary Information.
